# Kinetic Access to Focal Conic Domains in Photonic Cellulose Nanocrystal Films

**DOI:** 10.1021/acsnano.6c18491

**Published:** 2026-10-23

**Authors:** Diogo V. Saraiva, Lotte Polling, Sander J. W. Vonk, Samaneh Rahbar, Ivo R. Vermaire, Djouke A. C. Elferink, Rae M. Robertson-Anderson, Freddy T. Rabouw, Lisa Tran

**Affiliations:** † Debye Institute for Nanomaterials Science, 8125 Utrecht University, 3584 CC Utrecht, The Netherlands; ‡ Department of Physics and Biophysics, 7119 University of San Diego, San Diego, California 92110, United States; § Optical Materials Engineering Laboratory, Department of Mechanical and Process Engineering, 27219 ETH Zurich, Zurich 8092, Switzerland

**Keywords:** liquid crystals, cellulose nanocrystals, colloidal self-assembly, defects, structural color

## Abstract

Cellulose nanocrystals (CNCs) self-assemble into cholesteric structures that enable sustainable photonic films, but controlling their pitch, domain morphology, and angular color response remains challenging. Here, we investigate how salt concentration and sonication dose jointly regulate the evaporation-induced self-assembly of CNC suspensions. Across 24 dish-cast films, polarized optical microscopy, time-lapse imaging, rheology, electron microscopy, and angle-resolved spectroscopy reveal that these processing parameters affect not only the cholesteric pitch but also tactoid nucleation, transport, coalescence, and the onset of kinetic arrest. Increasing NaCl concentration generally blue-shifts the photonic response, whereas sonication red-shifts it by fragmenting CNC bundles, with the magnitude of this effect decreasing as ionic content increases. At intermediate ionic content, low sonication doses produce extended cholesteric domains with comparatively narrow spectral responses, while the abundance of focal conic domains (FCDs) increases with sonication dose, culminating in FCD-rich films at moderate sonication. Refined parameter scans show that FCDs form within a finite processing window. Time-lapse imaging shows that FCDs appear abruptly after tactoid growth and coalescence, as macroscopic motion becomes strongly restricted during late-stage evaporation. Concentration- and residence-time-dependent rheology further demonstrates that the material response evolves throughout the assembly pathway. The FCD-rich film retains a narrow local reflection band while exhibiting the smallest angular displacement of the reflection maximum among the films examined. These findings show that salt and sonication regulate the kinetic accessibility of elastically driven focal conic structures, providing a route to tune domain morphology, spectral bandwidth, and iridescence in CNC photonic films.

## Graphical abstract

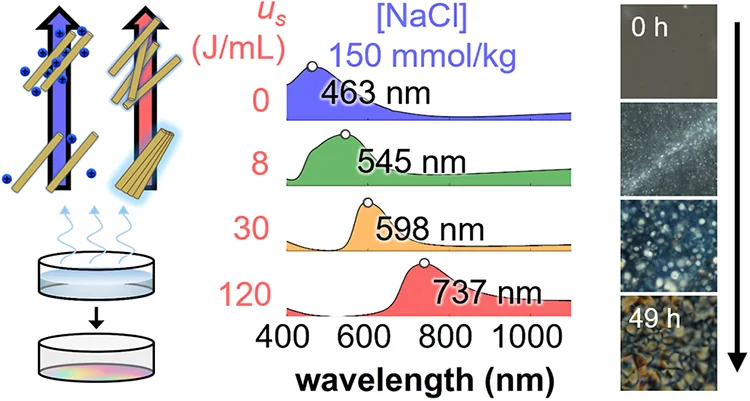

Structural color, originating from the interference between reflections of light on periodic or quasi-periodic nanostructures, has emerged as a promising mechanism for producing vibrant, pigment-free coloration. Its potential for use in sustainable coatings, optical sensors, and anticounterfeiting technologies has stimulated extensive research interest.^1–3^ Cellulose, an abundant and renewable biopolymer derived from plant biomass, offers a sustainable platform for the fabrication of structurally colored materials. The crystalline domains of cellulose fibrils can be isolated via acid hydrolysis to yield cellulose nanocrystals (CNCs), rod-like nanoparticles a few nanometers in width and hundreds of nanometers in length. When dispersed in water at appropriate concentrations, CNCs spontaneously self-assemble into a cholesteric liquid crystalline phase, whose helicoidal structure can be preserved upon solvent evaporation, producing iridescent solid films.^4^

The structural periodicity is quantified by the pitch (*p*), defined as the distance required for the CNC director to complete a 2π rotation (Figure 1a, right inset). For a locally planar cholesteric domain, the central selective-reflection wavelength is approximated by

1 $$\lambda =\mathrm{n̅}p\cos\theta_{\mathrm{int}}$$

where *n̅* is the average refractive index and θ_int_ is the angle between the wavevector inside the film and the local helical axis.^5,6^ For an external angle θ_air_ measured from the surface normal, Snell’s law gives

$$\lambda \left(\theta_{\mathrm{air}}\right)=p\sqrt{{\mathrm{n̅}}^{2}-n_{\mathrm{air}}^{2}\sin^{2}\theta_{\mathrm{air}}}.$$ 2

With *n*_air_ ≈ 1 and at normal incidence the relation reduces to λ = *n̅p*. Variations in pitch and local heli*x*-axis orientation therefore affect both the spectral and angular response of a CNC film.

**Figure 1:**
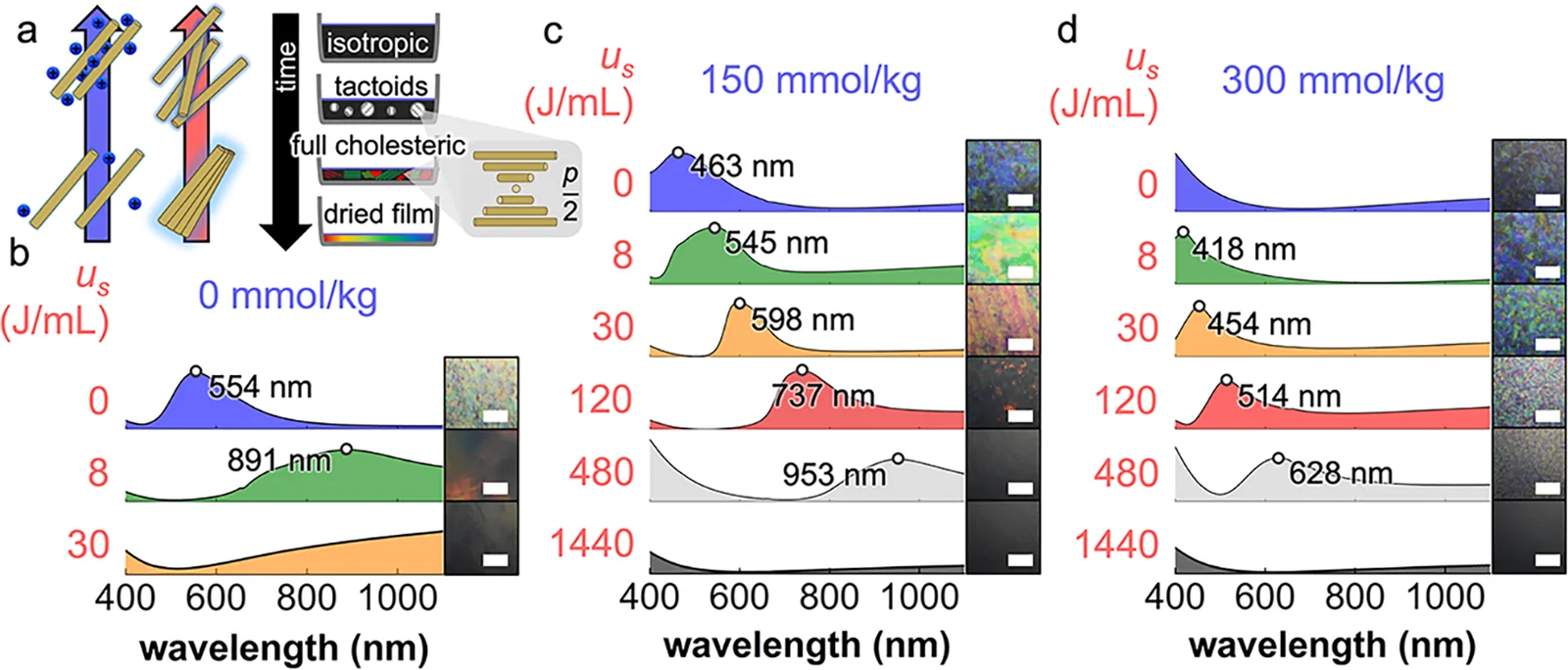
(a) Schematics illustrating how processing parameters influence CNC interactions and the resulting film color. Left: The blue arrow indicates that increasing salt concentration strengthens electrostatic screening, promoting closer CNC approach and blue-shifting the reflected color of the dried film. Center: The red arrow indicates that increasing sonication dose fragments CNC bundles, red-shifting the reflected color. Right: Schematics of a CNC suspension evaporating in a Petri dish with time progressing from top to bottom, leading to cholesteric self-assembly (with a half-pitch rotation of the CNCs depicted in the gray inset) and structural coloration in the final film. (b–d) Arrays of reflection-mode optical micrographs (captured with white light illumination through a left circular polarizer), each paired with a reflection spectrum from the same region. λ_max_ is reported for each spectrum, denoted by a white dot. Panels are ordered by NaCl concentration: (b) 0 mmol/kg of CNC, (c) 150 mmol/kg, and (d) 300 mmol/kg. Within each panel, spectra and images are arranged from top to bottom by sonication dose *u*_s_ (0–1440 J/mL). Scale bar: 200 μm.

Evaporation-induced self-assembly of CNCs, typically performed in confined geometries such as Petri dishes, proceeds through tactoid nucleation, coalescence, and eventual solidification,^7^ depicted schematically in Figure 1a (right). The fully dried film consists of multiple cholesteric domains delineated by topological defects that originate during the coalescence of tactoids.^8^ Each domain is characterized by a specific pitch, orientation, and lateral extent, collectively determining the film’s photonic response. Historically, optimization of CNC-based photonic materials has focused on promoting structural uniformity and minimizing defects to achieve narrow reflection bandwidths and high color saturation.^9^ Such uniformity has been pursued through strategies including reduction of CNC polydispersity,^10^ modulation of solvent evaporation rate,^7^ and application of shear to control helical alignment.^11,12^

While these approaches enhance structural order, they inherently preserve the angular dependence of the reflected color (eqs 1 and 2). Iridescence (angle-dependent color) remains intrinsic to the cholesteric architecture, limiting the use of CNC films in applications that require a consistent appearance over wide viewing angles. Recent studies have highlighted that the deliberate introduction of controlled disorder in a photonic system can suppress angular color dependence.^13,14^ Controlled disorder, manifested through particular spatial variations in domain orientation and defect distribution can induce diffuse light scattering that averages out directional reflection, thereby producing noniridescent structural color. Such effects are widely observed in biological photonic systems, where partial disorder and hierarchical organization yield angle-independent coloration.^15,16^ Consequently, the controlled manipulation of topological defects and domain heterogeneity in CNC assemblies presents a promising strategy to modulate photonic properties and achieve a uniform visual appearance.

Tuning the self-assembly pathway of CNCs through chemical and mechanical means enables control over the resulting structural hierarchy. The addition of electrolytes, such as sodium chloride (NaCl), screens the electrostatic repulsion between CNCs, strengthening chiral interactions and decreasing the pitch size, *p*, depicted schematically in Figure 1a (left).^17–20^ Conversely, ultrasonication disrupts CNC bundles that act as chiral dopants, thereby weakening chiral interactions and increasing *p* (Figure 1a, center).^21–23^ Both modifications influence multiple stages of evaporation-driven self-assembly, from tactoid nucleation and coalescence to kinetic arrest.^17,20–25^ Although the effects of salt and sonication on CNC viscosity have been individually reported,^26–28^ their combined influence and its implications for defect evolution and flow-induced ordering remain poorly understood.

In this work, we investigate how ionic content and sonication jointly regulate the evaporation-induced self-assembly of dish-cast CNC films. We prepare 24 films spanning a matrix of salt concentrations and sonication doses and characterize their color, birefringent texture, drying dynamics, rheological response, internal morphology, and angle-resolved reflection. The experiments reveal that salt and sonication affect not only the cholesteric pitch but also the sequence of tactoid nucleation, transport, coalescence, and the onset of kinetic arrest. Within a finite region of this processing space, these dynamics produce coherent cholesteric layers that subsequently develop toric and parabolic FCDs. By relating the assembly pathway to the final spectral bandwidth and angular response, we show how kinetic control of domain development provides access to photonic structures ranging from extended planar cholesteric domains to defect-rich films.

## Results and Discussion

### Tuning Color with Salt and Sonication

To investigate the effects of salt and sonication on the optical appearance of CNC films, we evaporated 24 CNC suspensions while tuning only their salt concentration, [NaCl] (four values from 0 up to 450 mmol/kg of CNC), and their sonication dose, *u*_s_ (six values from 0 up to 1440 J/mL). The CNCs used in this study are obtained from the acid hydrolysis of filter paper made from cotton, as detailed in the Methods section. The resulting CNC films exhibited photonic responses spanning from the UV to the IR. In Figure 1, we present micrographs of the films alongside their corresponding UV–vis attenuation spectra, calculated as log­(1/*T*), each with the peak wavelength λ_max_ labeled. Reflection micrographs and Petri dish photographs of the films are provided in SI Figures S1 and S2.

As expected, increasing the sonication dose *u*_s_ leads to an increase in λ_max_, whereas increasing [NaCl] results in a decrease in λ_max_, as shown in the reflection spectra in Figure 1. In the absence of added salt, raising *u*_s_ from 0 to 30 J/mL rapidly shifts λ_max_ into the infrared (Figure 1b). With increasing salt concentration, from [NaCl] = 150 to 300 mmol/kg, the red-shift of λ_max_ occurs more steadily, but an excessive sonication dose of 1440 J/mL eliminates any discernible λ_max_, as shown at the bottom of Figure 1c,d.

Overall, the data in Figure 1 indicate that increasing salt concentration diminishes the effectiveness of sonication in red-shifting the film’s photonic response. At [NaCl] = 0 mmol/kg, the range of λ_max_ spans more than 550 nm for 0 ≤ *u*_s_ ≤ 30 J/mL (Figure 1b), while a salt concentration of [NaCl] = 150 mmol/kg results in an intermediate λ_max_ range of about 430 nm for 0 ≤ *u*_s_ ≤ 480 J/mL (Figure 1c). At [NaCl] = 300 mmol/kg, the range narrows to approximately 250 nm for 0 ≤ *u*_s_ ≤ 480 J/mL (Figure 1d). Thus, sonication is most effective at red-shifting the photonic response under low-salt conditions and becomes progressively less effective as salt concentration increases.

This trend can be explained by the combined physical effects induced by salt and sonication. Increasing salt concentration increases ionic strength, strengthens electrostatic screening, and decreases the effective interaction range between CNCs. The CNCs possess an intrinsic sulfate half-ester content of 221 mmol kg^–1^ CNC, which gives the particles a substantial negative surface charge and contributes to electrostatic repulsion and colloidal stabilization. The addition of NaCl increases ionic screening, thereby weakening these repulsive interactions and allowing closer particle approach, while sufficiently high ionic content can promote aggregation. Because the fraction of CNC-associated counterions that remains mobile in suspension is not known, conductivity measurements are used as a comparative measure of the ionic environment experienced by the particles (SI Table S1).

Meanwhile, ultrasonication modifies the CNC size distribution by disrupting larger bundles and reducing the characteristic size of the dispersed particles. Atomic force microscopy (AFM) measurements show a progressive decrease in the projected length of deposited CNC particles with increasing *u*_s_, while dynamic light scattering (DLS) measurements show a corresponding decrease in apparent hydrodynamic size under dilute conditions (SI Figures S3 and S4). The AFM and DLS measurements together are interpreted qualitatively as evidence that sonication reduces the characteristic particle and bundle size. Fragmentation increases the number of independently dispersed objects and modifies their effective interaction volume and number-density. In low-salt media with a longer electrostatic interaction range, this increase in the number of dispersed particles can reduce packing efficiency and contribute to a red-shift in pitch. In contrast, stronger screening at higher salt concentration reduces the effective interaction range and allows the CNCs to pack more closely into the cholesteric structure, diminishing the sonication-induced red-shift.^23^

### Effect on Film Morphology

In addition to affecting the cholesteric pitch, salt and sonication also strongly influence the final film morphology. To examine these effects, we used polarized optical microscopy (POM) to observe films formed from solutions with varying sonication doses and salt concentrations. Figure 2 displays all film morphologies in a 6 × 4 grid of transmission images taken between crossed polarizers.

**Figure 2:**
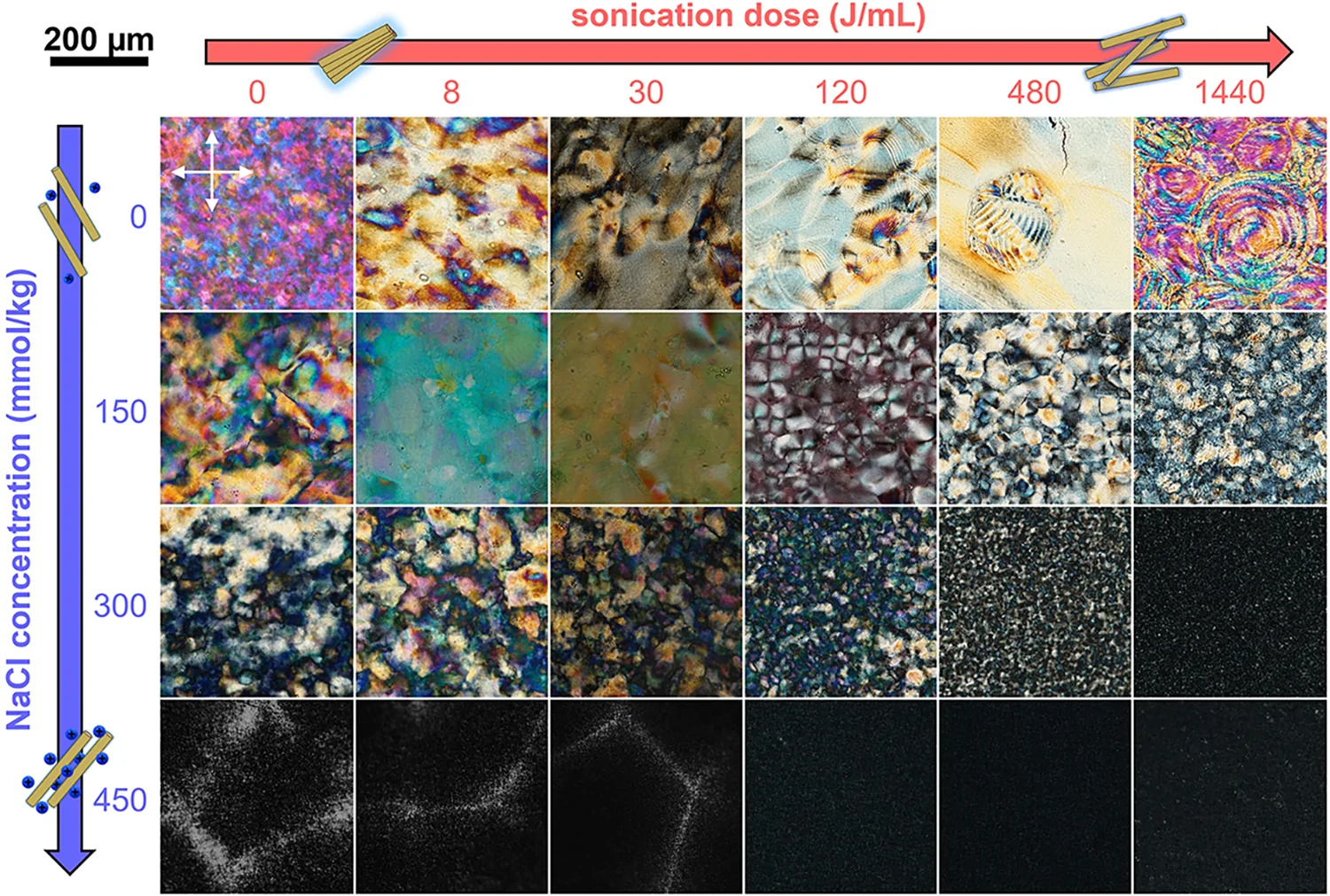
An array of micrographs of CNC films taken in transmission mode between crossed polarizers. Bright regions exhibit detectable birefringence and therefore contain anisotropically ordered structures, whereas dark regions exhibit little detectable birefringence under these measurement conditions. The CNC films were made from suspensions prepared with varying NaCl concentrations (increasing from top to bottom) and sonication doses (increasing from left to right). Each image was taken at the center of its respective film (within a 2 mm radius). The scale bar for all images is displayed in the top-left corner.

The cast films vary considerably in texture, particularly in domain size and density. In films with a salt concentration of 450 mmol/kg, regions that are dark under crossed polarizers dominate, and only a few birefringent domains are retained, indicating that little long-range birefringent order is retained in these regions. Surprisingly, these small birefringent domains are organized into lines. For 300 and 450 mmol/kg, increasing sonication dose gradually reduces the size of the domains. At low salt concentrations (0 and 150 mmol/kg), more bright, birefringent regions are observed under crossed polarized microscopy, suggesting that (regardless of color) cholesteric structure is more readily obtained at these solution conditions, although the domain sizes and shapes vary widely. Notably, at 150 mmol/kg, films prepared at sonication doses of 8 and 30 J/mL contain large, extended cholesteric regions, while closer inspection also reveals Maltese-cross textures, which we identify as focal conic domains (FCDs), regions of cholesteric “layers” (defined by the half-pitch) that curve around focal defect lines.^29–31^ FCDs are sparse at 8 J/mL, become more frequent at 30 J/mL (see the top right of the micrograph in Figure 2), and are prominent throughout the film at 120 J/mL, where characteristic Maltese-cross textures are readily observed.

These observations reflect how salt concentration influences the onset of gelation and the development of cholesteric ordering. From previous studies, it is known that increasing salt strengthens electrostatic screening, lowering the CNC concentration at which kinetic arrest occurs while raising the critical CNC concentration for cholesteric nucleation.^20,32^ The predominance of optically dark regions at 450 mmol/kg is consistent with structural rearrangement becoming restricted before extensive cholesteric order develops. At 300 mmol/kg, the greater extent of birefringent structure is consistent with structural rearrangement remaining possible for longer, allowing more cholesteric order to develop before mobility becomes strongly restricted. At 150 and 0 mmol/kg, a larger fraction of the cholesteric structure is retained when macroscopic mobility becomes restricted.

When considering the effect of ultrasonication, films with [NaCl] = 300 and 450 mmol/kg show a decrease in average domain size with increasing sonication dose. This reduction may be associated with increases in the concentrations at which the cholesteric phase nucleates, forming birefringent tactoids, and at which the system is fully cholesteric as sonication increases.^23^ In addition, the suspension may gel at earlier stages of tactoid formation,^22^ due to sonication fragmenting CNC particles and bundles.

At the higher salt concentrations, the decrease in domain size with increasing sonication dose *u*_s_ suggests insufficient time for tactoid annealing. Sonication-induced fragmentation disrupts the formation of cholesteric order, while early gelation further restricts structural development. Consequently, higher sonication doses under these salt conditions lead to smaller cholesteric domains.

At lower [NaCl], the influence of sonication is more complex. Without added salt, increasing *u*_s_ produces larger birefringent regions with fewer visible domain boundaries. At 150 mmol/kg, films prepared at *u*_s_ = 8 and 30 J/mL contain extended, comparatively uniform cholesteric regions and have relatively narrow spatially averaged spectral responses (SI Table S2). These films also contain FCDs, with sparse FCDs at 8 J/mL and a greater FCD abundance at 30 J/mL. At *u*_s_ = 120 J/mL, FCDs become a dominant feature of the morphology, producing the FCD-rich film examined in detail below. The 150 mmol/kg series therefore shows a progressive increase in FCD abundance with sonication dose, from predominantly extended cholesteric regions containing occasional FCDs to an FCD-rich morphology.

These observations suggest that the final morphology depends on the interval over which tactoids can nucleate, move, and coalesce before structural rearrangement becomes strongly restricted. Salt and sonication shift both the development of cholesteric order and the mobility of the concentrated suspension. The following sections examine the FCD morphology and the associated drying pathway in greater detail.

### Focal Conic Domains

To investigate the self-assembly process leading to the FCD-rich morphology, we recorded a time-lapse of film evaporation for the [120 J/mL, 150 mmol/kg] condition using POM. Figure 3 presents selected frames from this time-lapse (Figure 3a–e; full sequence provided in SI Video 1), together with a scanning electron micrograph of the film cross-section (Figure 3f), a POM image of parabolic FCDs formed under a nearby processing condition (Figure 3g), and three-dimensional schematics of the toric and parabolic focal conic geometries (Figure 3h,i, respectively).

**Figure 3:**
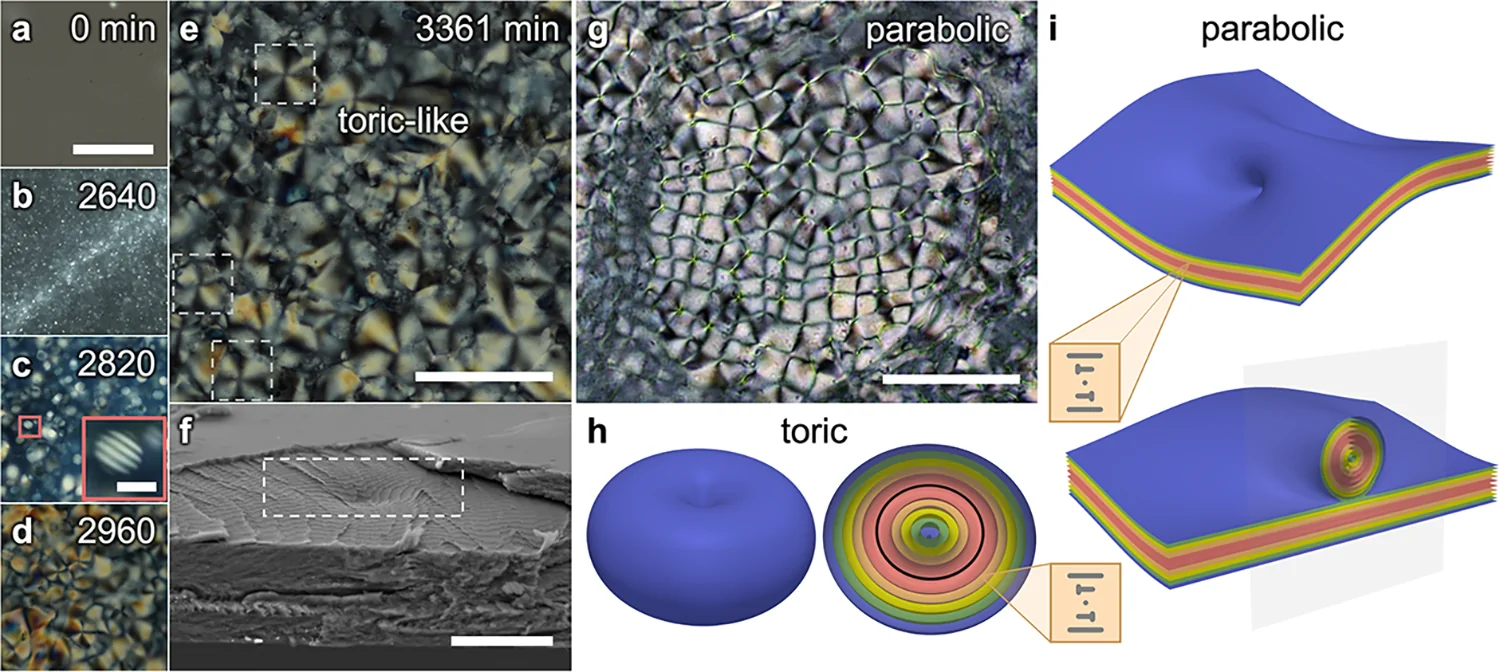
(a–e) Frames from the time-lapse video of a [120 J/mL, 150 mmol/kg] CNC suspension during evaporation. (a: 0 min) Evaporation begins with the solution in the isotropic regime; (b: 2640 min) the critical cholesteric concentration is reached, tactoids nucleate and organize into lines; (c: 2820 min) tactoids grow and coalesce, a tactoid is highlighted in the solid red inset (scale bar: 10 μm); (d: 2960 min) macroscopic motion becomes undetectable; (e: 3361 min) the film is compressed as evaporation is completed. Toric-like FCDs are highlighted with gray dashed squares. (f) SEM image of the film cross-section and exposed glass-air interface. (g) POM image of a film made from a [120 J/mL, 100 mmol/kg] CNC suspension exhibiting a square array of parabolic FCDs. (h) Idealized schematics of a toric FCD (cross section, right; the circular focal line is plotted in black). (i) Schematics of a parabolic FCD (cross section, bottom). (h, i) Each colored layer plotted represents a half-pitch rotation of the cholesteric, represented in the orange highlight with a cholesteric nail diagram. Scale bars: (a–e, g) 200 μm, (c) 30 μm.

The idealized toric and parabolic schematics in Figure 3h,i connect the observed optical textures to their three-dimensional layer geometries. The toric structure consists of nested layers surrounding circular and axial focal lines,^33,34^ whereas the parabolic structure is formed from nested parabolic Dupin cyclides.^35^ In both representations, the local helical axis is normal to the cholesteric layers.

Initially, the CNC suspension is isotropic (Figure 3a, 0 min). After several hours of gradual evaporation, tactoids begin to nucleate and align into linear arrangements (Figure 3b, 2640 min). As evaporation progresses, the cholesteric tactoids, identifiable from their characteristic fingerprint texture, grow and coalesce, as shown in Figure 3c at 2820 min, with a tactoid highlighted in the inset. At around 2960 min, macroscopic flow visibly slows and eventually ceases. Note that this visual halt cannot be conclusively attributed to kinetic arrest. Instead, it may reflect restricted mobility caused by a high volume fraction of cholesteric phase, which is known to have long relaxation times.^36^ Immediately following this event, FCDs emerge rapidly throughout the film. Afterward, only minimal structural changes are observed.

The 4-fold Maltese-cross textures that appear during the final stages of the time-lapse are consistent with approximately axisymmetric focal conic structures. Their optical appearance resembles the ideal toric limit illustrated in Figure 3h, in which nested layers curve around circular and axial focal lines.^33,34^ However, the experimentally observed domains vary in size and packing, and the SEM images reveal an irregular network of curved and undulating layers. In particular, the egg-crate-like topography in the SEM imaging of the film cross section in Figure 4 contains neighboring dome-like and saddle-like regions, consistent with the presence of both locally positive and negative Gaussian curvature within the cholesteric layered structure. This mixed-curvature morphology, together with the Maltese-cross texture, supports an approximately toric organization. We therefore describe these structures as toric-like FCDs, for which only part of the corresponding ideal focal-conic construction may be physically contained within the film, while other portions are truncated by the film boundaries and remain virtual.^37^ The toric schematic represents an idealized geometric reference.

**Figure 4:**
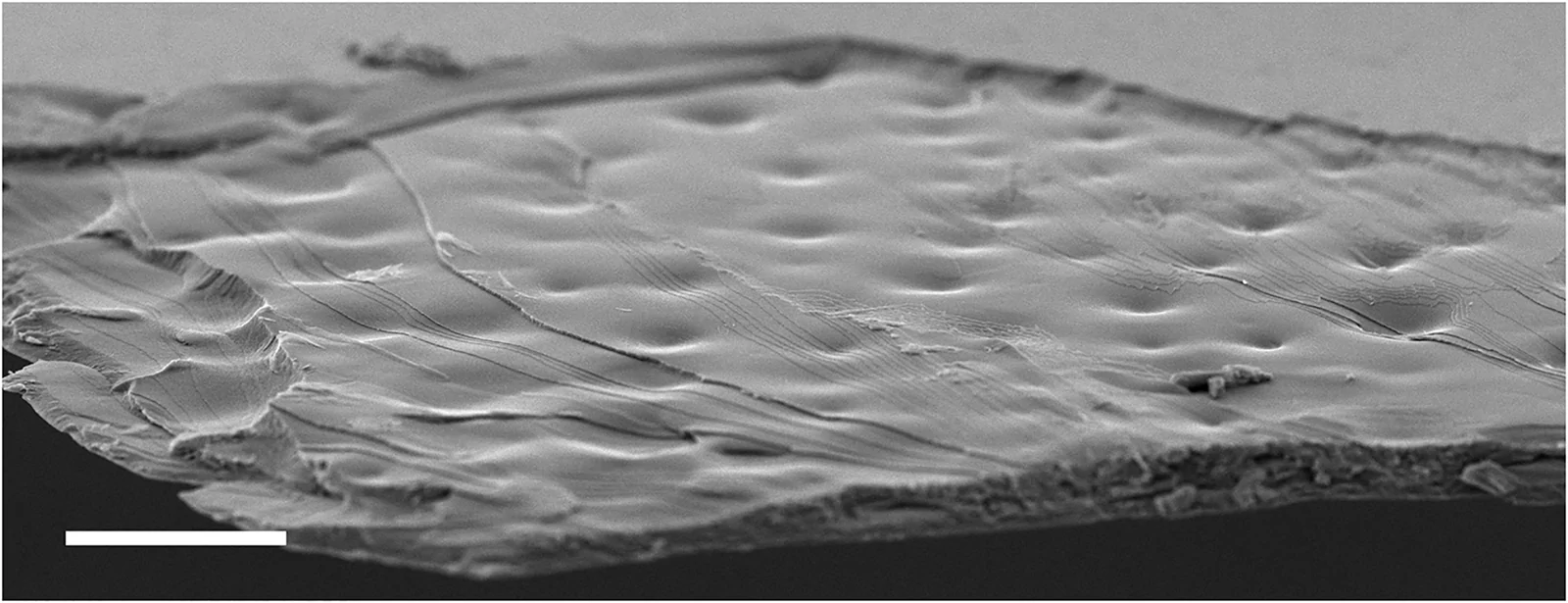
SEM image of the cross-section and exposed glass-air interface of the [120 J/mL, 150 mmol/kg] film, showing multiple concavities indicative of FCDs. Scale bar: 100 μm.

In addition to these toric-like FCDs, the finer salt-concentration scan revealed more regularly arranged parabolic FCD textures. Figure 3g shows a representative POM image from a [120 J/mL, 100 mmol/kg] film, selected from the complete fixed-sonication salt series presented in SI Figure S5. The approximately square arrangement of the optical texture is consistent with interacting parabolic Dupin cyclides and their associated focal lines, represented schematically in Figure 3i.^35^ The observations therefore indicate that the FCD morphology varies across the broader FCD-forming processing window, ranging from irregular toric-like domains with potentially substantial virtual portions to more regularly arranged parabolic textures.

The coarse grid in Figure 2 identified [120 J/mL, 150 mmol/kg] as an FCD-rich condition. Closer inspection of the 150 mmol/kg series shows that FCD formation is not restricted to this sonication dose. Sparse FCDs are present at *u*_s_ = 8 J/mL (SI Video 4), become more frequent at 30 J/mL (SI Video 5), and are prominent throughout the film at 120 J/mL (SI Video 1). Thus, over this low-to-moderate sonication range, increasing *u*_*s*_ increases the abundance of FCDs while the surrounding cholesteric morphology also evolves.

The refined parameter scans further show that FCD formation extends over a broader region of salt concentration and sonication dose. At *u*_s_ = 120 J/mL, FCDs are absent at 0–50 mmol NaCl/kg CNC and appear from 75 to 150 mmol/kg (SI Figure S5). A two-dimensional scan over *u*_s_ = 60–180 J/mL and [NaCl] = 75–225 mmol/kg further identifies an intermediate-salt region containing prominent FCDs (SI Figure S6). The [120 J/mL, 100 mmol/kg] film in Figure 3g provides a representative parabolic FCD texture from this refined scan. FCD formation at [120 J/mL, 150 mmol/kg] was also reproduced using CNCs from three independent hydrolysis batches (SI Figure S7).

FCDs have previously been observed in CNC systems, both in capillaries^38^ and in evaporated films with pitch values of approximately 3 μm.^31^ Although the mechanism of FCD formation was not fully explored in those works, it has been proposed^39^ that compression-induced pitch contraction may trigger a Helfrich-Hurault-like instability, whereby a mismatch between the preferred layer spacing and the constrained structure drives undulation of the cholesteric layers.^40–43^ In capillaries, FCDs appeared after one year of storage and developed further upon heating to 60 °C for 1 week. Both processes caused mild CNC desulfation, which reduced surface charge and contracted the cholesteric pitch.^39^

Compared with these previous reports, our system is distinct. FCDs form within a finite region of salt concentration and sonication dose and have submicron pitch values that give rise to visible-to-near-infrared selective reflection. SEM imaging of the film cross-section (Figure 3f) reveals curved and undulating layers within the interior, while a second region shows an egg-crate-like surface topography composed of neighboring concave and convex features (Figure 4). These structures span approximately 10–100 μm, comparable to the lateral dimensions of the Maltese-cross domains observed by POM in Figure 3e.

The schematics in Figure 3h,i provide idealized geometric references for relating these optical textures to possible three-dimensional layer arrangements. The toric construction illustrates how nested, approximately axisymmetric layers can generate a Maltese-cross texture. The experimentally observed domains exhibit a less regular geometry, consistent with the mixed dome-like and saddle-like curvature evident in the surface topography and with truncation of the focal-conic construction by the finite film boundaries.^37^ The toric schematic therefore captures the characteristic layer curvature and symmetry of these toric-like domains. The parabolic construction provides the corresponding geometric reference for the more regularly arranged, approximately square focal-conic textures observed in Figure 3g, in which nested parabolic Dupin cyclides are organized around their focal lines.

The POM time series and SEM morphology place FCD formation at a late stage of the drying process, after extended cholesteric regions have already developed through tactoid growth and coalescence. The abrupt appearance of the Maltese-cross textures and the curved, undulating layers retained in the dried film are consistent with a collective deformation of this pre-existing layered structure. A Helfrich-Hurault-like layer-undulation mechanism provides a plausible physical framework for this transformation.^40–43^ These observations establish the sequence followed by the FCD-rich [120 J/mL, 150 mmol/kg] condition and motivate comparison with other processing conditions to determine which features of the drying pathway are associated with access to FCD formation.

### Comparison of Self-Assembly Pathways during Evaporation

We compared time-lapse recordings across the processing space to examine how cholesteric assembly and tactoid transport vary with salt concentration and sonication dose. Figure 5 shows representative sequences for [1440 J/mL, 0 mmol/kg], [8 J/mL, 150 mmol/kg], and [0 J/mL, 450 mmol/kg] (SI Videos 7, 4, and 8), complementing the FCD-rich [120 J/mL, 150 mmol/kg] sequence discussed above. Each suspension begins in the isotropic regime and subsequently develops cholesteric tactoids that grow and coalesce into larger birefringent regions before visible texture motion becomes strongly reduced. The onset and duration of these stages vary substantially among the processing conditions.

**Figure 5:**
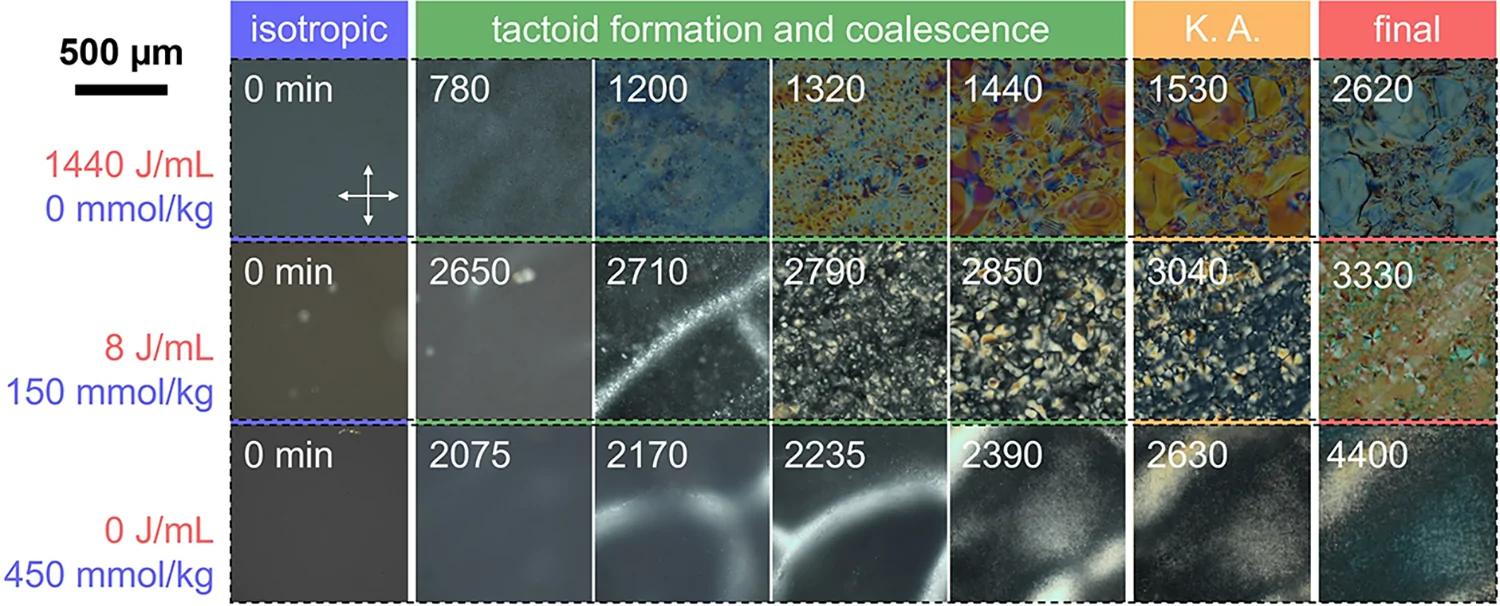
Frames taken from three time-lapse videos of evaporating CNC suspensions: [1440 J/mL, 0 mmol/kg], [8 J/mL, 150 mmol/kg], [0 J/mL, 450 mmol/kg]. All time-lapse videos begin in the isotropic regime at [CNC] = 3 wt %. Tactoid formation and coalescence follow, and convection-like flow patterns are observed in the bottom two samples. The second-to-last frame (K.A., kinetic arrest) marks the point at which tactoid motion ceases; however, this frame does not represent the exact moment of kinetic arrest, which cannot be determined visually as a lack of texture evolution can also arise from a high volume fraction of cholesteric phase. The final frame corresponds to complete evaporation. The scale bar for all frames is shown on the top left.

The final films in Figure 2 retain structural motifs resembling different stages of these drying trajectories. Predominantly nonbirefringent films at high salt concentration and high sonication dose resemble the early stage before extensive cholesteric order develops, whereas the linear birefringent structures retained at high salt concentration and low sonication dose resemble tactoid arrangements formed during the biphasic stage. At 300 mmol/kg, the progression from smaller birefringent regions at high sonication dose to larger cholesteric regions at lower sonication dose similarly reflects differences in tactoid growth and coalescence. These observations indicate that the processing conditions alter both the progression of cholesteric assembly and the organization of the tactoids during drying.

Beyond these differences in structural evolution, the time-lapse sequences reveal two distinct large-scale modes of tactoid transport. All samples exhibit radial motion toward the Petri-dish perimeter, consistent with capillary flow generated by the higher evaporation rate near the contact line.^44^ This outward transport contributes to the coffee-ring effect and the radial color gradients observed in the films (SI Figure S2). A second mode of motion accompanies the formation of linear tactoid arrangements, visible at approximately 2710 min for [8 J/mL, 150 mmol/kg] and 2170 min for [0 J/mL, 450 mmol/kg] in Figure 5. These bands develop within regions showing apparent recirculating motion, consistent with buoyancy-associated transport during isotropic-cholesteric phase separation. Tactoids accumulate along the boundaries of the circulating regions and subsequently disperse as continued motion and coalescence reorganize the cholesteric structure. We attribute these convection-like recirculating patterns to buoyancy-associated transport during isotropic-cholesteric phase separation. They were observed in the samples containing added salt, whereas the salt-free samples showed predominantly radial outward motion.

These transport modes also differ in their spatial organization. The radial motion extends broadly across the field of view, whereas the convection-like motion is localized into circulating regions separated by boundaries along which tactoids accumulate. The latter therefore produces pronounced spatial variation in the magnitude and direction of the observed motion. Together with the concurrent tactoid growth, deformation, and coalescence, this complexity makes visual inspection alone insufficient to compare the dynamics across processing conditions and motivates the quantitative analysis presented in the following section.

To isolate the influence of the drying pathway on tactoid assembly, we evaporated another [120 J/mL, 150 mmol/kg] suspension under the same composition, substrate, and nominal anchoring conditions as Figure 3, but with the Petri dish partially covered. The full time-lapse is provided in SI Figure S8 and SI Video 2. Slowing evaporation from approximately 2 to 11 days suppressed visible capillary and convection-like motion. The linear tactoid bands observed during standard drying were also absent throughout the slow-evaporation sequence. The suspension instead self-assembled into numerous small tactoids that remained spatially dispersed and did not fully coalesce, producing a domain-wall-rich structure instead of the FCD-rich morphology. Because salt concentration, sonication dose, substrate, and nominal anchoring were held fixed, this comparison shows that evaporation rate and the associated transport dynamics influence tactoid organization and coalescence and thereby alter the final film morphology.

The visual time-lapse comparison therefore identifies differences in both the timing and spatial character of the assembly dynamics across the processing parameters. The time-lapse observations show that salt concentration, sonication dose, and evaporation rate alter both the progression of cholesteric assembly and the spatial organization of tactoid motion and coalescence during drying.

### Time-Resolved Image Analysis of Self-Assembly Dynamics

To quantify how salt concentration and sonication dose alter the timing and spatial organization of cholesteric assembly, we analyze the POM time-lapses using BARCODE, a time-resolved image-analysis platform that couples image binarization with optical-flow analysis.^45,46^ This approach tracks the evolution of tactoid population and domain size together with the magnitude and spatial heterogeneity of the image dynamics, allowing tactoid nucleation, growth and coalescence, and the later FCD-associated rearrangement to be compared across processing conditions.

Image binarization identifies connected birefringent regions, referred to here as islands, which during the early stages of assembly approximately represent disconnected tactoid or domain regions. The number of islands therefore reports the population of disconnected optical regions, while the mean island area characterizes their average spatial extent. Optical-flow analysis determines frame-to-frame changes in the image texture. The mean speed is the spatially averaged magnitude of the optical-flow vectors across the field of view and includes contributions from tactoid translation, growth, deformation, and coalescence. The speed standard deviation (speed SD) measures the spread in optical-flow speeds across the image and therefore reports the spatial heterogeneity of the dynamics. The time evolution of these four metrics, number of islands, mean island area, mean speed, and speed SD, is shown for the complete POM time-lapses of the 150 mmol/kg series in SI Figure S9. Because all four quantities are image-derived, they are interpreted together with the corresponding POM textures.

Figure 6a illustrates how the image-analysis outputs correspond to three stages of the FCD-forming [120 J/mL, 150 mmol/kg] trajectory. At initial cholesteric nucleation, numerous small birefringent tactoids appear as disconnected islands in the binarized image, while the optical-flow field contains relatively small velocity vectors of similar magnitude. During subsequent tactoid growth and coalescence, the islands become larger and the optical-flow field shows faster and more spatially heterogeneous motion. At FCD onset, Maltese-cross textures appear within the extended cholesteric regions (circled in Figure 6a). Large-scale tactoid transport is nearly halted by this stage, while localized optical-flow activity accompanies the development of the FCD texture. At this stage, the optical-flow signal reflects the local structural reorganization associated with formation of the Maltese-cross texture. Similarly, island number no longer corresponds directly to the number of physical tactoids because the alternating bright and dark sectors of a single FCD can generate multiple disconnected islands after binarization.

**Figure 6:**
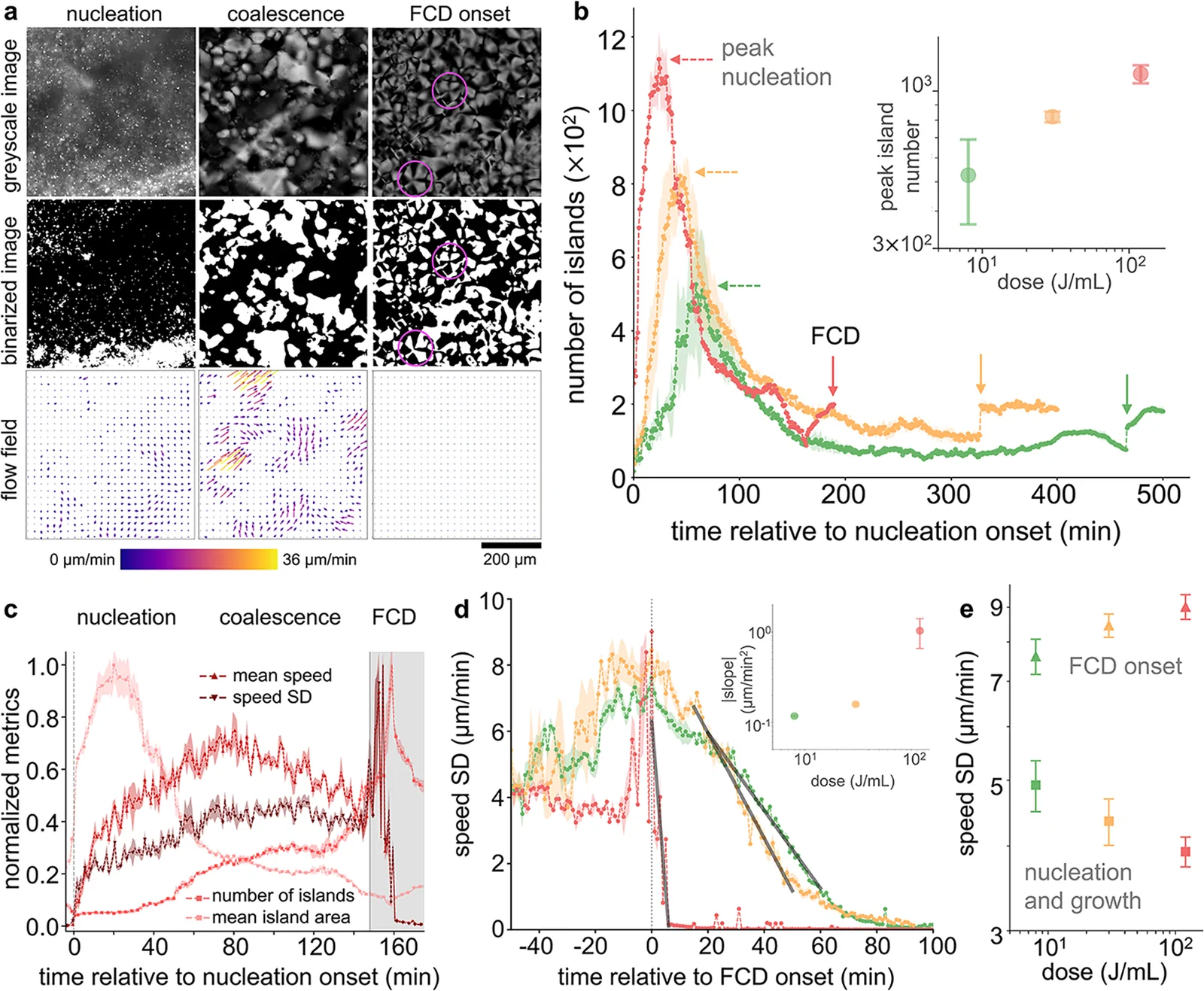
Time-resolved image analysis of the pathway leading to FCD formation. (a) Representative grayscale POM images, corresponding binarized “island” images, and optical-flow fields for the [120 J/mL, 150 mmol/kg] suspension during initial cholesteric nucleation, subsequent tactoid growth and coalescence, and the first clear appearance of FCDs. Magenta circles highlight representative Maltese-cross FCD textures and their corresponding binarized islands. (b) Time evolution of island number for the 8, 30, and 120 J/mL samples at 150 mmol/kg, plotted relative to the first persistent appearance of cholesteric tactoids. Dashed arrows mark the initial tactoid-associated maxima and solid arrows mark the later FCD-associated increases. The inset shows the maximum tactoid-associated island number as a function of sonication dose. (c) Time evolution of four BARCODE metrics for the [120 J/mL, 150 mmol/kg] condition: number of islands, mean island area, mean speed, and speed standard deviation (SD). Each metric is normalized to its individual maximum to compare their relative timing. Dashed and solid vertical lines indicate the onset of cholesteric nucleation and FCD formation, respectively. (d) Time evolution of speed SD for 8, 30, and 120 J/mL, shifted to align their FCD-associated peaks at *t* = 0. Solid lines show linear fits to the postpeak decay, with the corresponding slope magnitudes shown in the inset. (e) Speed SD during the initial nucleation/growth stage and at FCD onset as a function of sonication dose. Increasing sonication produces more spatially homogeneous dynamics during initial tactoid assembly and greater spatial heterogeneity at FCD onset.

To determine how the assembly pathway changes with FCD abundance, Figure 6b compares the time evolution of island number for the 8, 30, and 120 J/mL samples at fixed [NaCl] = 150 mmol/kg. Time is shifted such that *t* = 0 corresponds to the first persistent appearance of cholesteric tactoids. During the initial assembly stage, the maximum tactoid-associated island population increases from 8 to 30 to 120 J/mL, as highlighted by the dashed arrows and inset of Figure 6b. This maximum is also reached progressively faster with increasing sonication dose. The subsequent decrease in island number reflects the growth and coalescence of these initially disconnected regions. At later times, a second increase in island number appears for each FCD-forming condition and corresponds to the development of the FCD optical texture (solid arrows in Figure 6b). This FCD-associated response occurs progressively earlier relative to tactoid nucleation as sonication dose increases.

The unsonicated [0 J/mL, 150 mmol/kg] condition provides a same-salt comparison for this trend. It develops a substantial tactoid population and eventually forms large cholesteric domains, but its tactoid growth and coalescence proceed substantially more slowly than in the sonicated samples (SI Figure S10), and the final film retains a non-FCD cholesteric morphology. Across the 150 mmol/kg series, the progression from sparse FCDs at 8 J/mL to the FCD-rich morphology at 120 J/mL is therefore associated with increasingly rapid generation, growth, and coalescence of the tactoid population.

The time evolution of the complete FCD-forming pathway is resolved for [120 J/mL, 150 mmol/kg] in Figure 6c, where the four BARCODE metrics are normalized to their individual maxima to compare their temporal evolution. Immediately following nucleation, island number rises rapidly and mean island area increases as tactoids appear and grow. Island number subsequently decreases while mean island area continues to increase, consistent with coarsening and coalescence into fewer, larger cholesteric regions. Mean speed and speed SD increase during the initial period of nucleation and growth. As tactoid growth and coalescence continue, mean speed decreases while speed SD remains comparatively steady, showing that the overall rate of texture evolution decreases while substantial spatial heterogeneity in the motion persists.

The first clear appearance of the Maltese-cross texture is accompanied by a rapid increase in both mean speed and speed SD, followed by a sharp decrease in both quantities. Comparable mean-speed and speed-SD values are observed during tactoid nucleation and growth across both FCD-forming and non-FCD-forming conditions, whereas the rapid late increase and subsequent decrease are characteristic of the FCD-forming trajectories at 150 mmol/kg (SI Figure S9). This paired response therefore provides a dynamical marker of FCD onset and indicates a discrete, spatially heterogeneous structural rearrangement within the already developed cholesteric structure. The accompanying increase in island number partly reflects segmentation of the newly formed Maltese-cross texture into multiple bright regions, as illustrated in Figure 6a. Following this event, both mean speed and speed SD decrease rapidly and comparatively little further texture evolution occurs.

To examine how the dynamics of FCD onset vary with FCD abundance, the speed-SD trajectories for 8, 30, and 120 J/mL are shifted to align their FCD-associated peaks at *t* = 0 in Figure 6d. The peak speed SD increases systematically with sonication dose, indicating progressively greater spatial heterogeneity during FCD formation. The subsequent decay also becomes substantially faster with increasing sonication. The 8 and 30 J/mL samples retain heterogeneous motion for tens of minutes following the peak, whereas the [120 J/mL, 150 mmol/kg] condition loses this activity within only a few minutes. Linear fits to the postpeak region quantify this trend, with the corresponding slope magnitudes shown in the inset of Figure 6d.

Figure 6e summarizes the effect of sonication dose on the spatial distribution of the dynamics during the initial nucleation/growth stage and at FCD onset. During nucleation and growth, speed SD decreases from 8 to 30 to 120 J/mL, indicating progressively more spatially homogeneous dynamics. At FCD onset, speed SD instead increases with sonication dose, producing an increasingly pronounced separation between the early assembly dynamics and the late FCD-associated response. The FCD-rich 120 J/mL condition therefore combines comparatively homogeneous tactoid-scale assembly with a strongly heterogeneous late rearrangement. This inversion is consistent with rapid growth and coalescence of an extended cholesteric precursor followed by localization of the dynamics during the late collective layer deformation associated with FCD formation.

The unshifted trajectories in SI Figure S9 show the corresponding evolution in real drying time, while the event-aligned plots allow the sequence and relative timing of structural changes to be compared across conditions. Because solvent loss may be nonlinear and the liquid-layer geometry evolves continuously during evaporation, elapsed time cannot be converted directly into CNC concentration. Summary comparisons of the characteristic island populations and assembly times, including the non-FCD-forming [0 J/mL, 150 mmol/kg] control, are provided in SI Figure S10.

The time evolution of the four BARCODE metrics for the extreme [1440 J/mL, 0 mmol/kg] and [0 J/mL, 450 mmol/kg] conditions is compared with [8 J/mL, 150 mmol/kg] in SI Figure S11. Similar to the FCD-forming conditions, these extreme samples exhibit a finite period of increased structural development and dynamics, with metric values comparable in magnitude to those measured during tactoid growth in the FCD-forming samples. The major difference is temporal. The extreme conditions enter this active assembly regime much earlier in the drying process and subsequently evolve toward their final non-FCD morphologies, whereas the FCD-forming samples exhibit the additional rapid late response associated with FCD onset. Abundant FCD formation is therefore associated with cholesteric tactoid formation occurring sufficiently late in the drying process, followed by rapid tactoid growth and coalescence into an extended cholesteric precursor.

The controlled evaporation-rate experiment provides a complementary comparison at fixed salt concentration and sonication dose. Slower evaporation of the [120 J/mL, 150 mmol/kg] suspension suppresses visible capillary- and convection-like motion and produces a domain-wall-rich film. The corresponding BARCODE analysis is shown in SI Figure S12. Following tactoid formation, the slowly evaporated sample retains a larger number of islands with substantially smaller mean island areas over an extended interval, demonstrating slower and more prolonged tactoid growth and coalescence than during standard evaporation. The structural dynamics likewise evolve gradually throughout this interval, and the sharp late FCD-associated response characteristic of standard drying does not develop. The resulting film retains numerous incompletely coalesced tactoids and domain boundaries. This comparison is consistent with a kinetic-elastic picture in which rapid formation of an extended layered precursor enables the subsequent collective deformation associated with FCD formation during continued evaporation.

A possible extension of this interpretation concerns the phase-transition pathway during drying. Early tactoid formation is consistent with nucleation and growth within the isotropic-cholesteric coexistence region, while continued concentration could alter how the residual isotropic fraction develops cholesteric order. Zhu et al. proposed that CNC film formation can involve an early nucleation-and-growth stage followed by spinodal decomposition of the residual isotropic fraction.^47^ Such a transition could contribute to rapid development of a spatially continuous cholesteric precursor before FCD formation. The structural and dynamical trajectories described above are compatible with this possibility, although they do not distinguish spinodal decomposition from dense tactoid nucleation, growth, and coalescence.

The time-resolved image analysis therefore identifies a characteristic kinetic sequence associated with abundant FCD formation. Cholesteric ordering occurs relatively late in the drying process and is followed by rapid tactoid generation, growth, and coalescence into an extended layered precursor. Within the 150 mmol/kg series, increasing sonication accelerates this postnucleation evolution, whereas the [0 J/mL, 150 mmol/kg] condition follows a slower coarsening pathway toward large non-FCD cholesteric domains (SI Figure S10). The extreme [1440 J/mL, 0 mmol/kg] and [0 J/mL, 450 mmol/kg] conditions enter the structurally active regime substantially earlier in drying and evolve toward different final morphologies (SI Figure S11). FCD formation is subsequently marked by a discrete, spatially heterogeneous rearrangement within the already developed cholesteric structure.

Rheological measurements provide complementary information about how the material response evolves with CNC concentration and residence time (SI Figure S13). For the FCD-forming [120 J/mL, 150 mmol/kg] suspension, the initial and one-month flow curves remain comparatively similar at 4 and 6 wt % CNC, whereas a pronounced aging response develops at 8 wt % and remains evident at 10 wt %. The strong increase in low-shear apparent viscosity and enhanced shear thinning at 8 wt % are consistent with the development of a more persistent, shear-sensitive mesoscale organization. These aged measurements cannot be mapped onto the instantaneous viscosity of the drying film, but they establish that the same formulation that initially supports extensive tactoid rearrangement can develop a substantially more persistent structural response as concentration and residence time increase.

The rheological measurements show that the concentration dependence of the suspension response is strongly affected by sonication and residence time at fixed CNC concentration (SI Figure S13). For measurements performed approximately 3 days after preparation at the target concentration, the [0 J/mL, 150 mmol/kg] suspension remains comparatively low in apparent viscosity across the measured concentration range, whereas the FCD-forming [120 J/mL, 150 mmol/kg] suspension develops a progressively stronger response with increasing CNC concentration. After one month at fixed concentration, both formulations exhibit pronounced shear-sensitive responses, but at different concentrations. The [0 J/mL, 150 mmol/kg] suspension shows its strongest increase in low-shear apparent viscosity near 6 wt % CNC, whereas the [120 J/mL, 150 mmol/kg] suspension develops its strongest response near 8 wt %. This nonmonotonic concentration dependence indicates that sonication changes the concentration range over which persistent, shear-sensitive mesoscale organization becomes rheologically apparent. AFM and DLS further show that the unsonicated suspension contains a larger characteristic particle and bundle population (SI Figures S3 and S4), while removal of part of this large-object population by filtration increases the area occupied by uniform cholesteric domains (SI Figure S15). These results indicate that sonication shifts the concentration-dependent rheological response while reducing the characteristic particle and bundle size, changes that correlate with more rapid tactoid growth and coalescence and with access to the FCD-forming pathway at 150 mmol/kg.

These kinetic, structural, and rheological differences suggest that access to FCD formation depends on how rapidly an extended cholesteric precursor develops as structural rearrangement becomes progressively restricted. Continued evaporation increases CNC concentration, contracts the preferred cholesteric pitch, and decreases the film thickness. Once an extended layered structure has formed, continued compression may generate elastic strain as its capacity for structural relaxation diminishes. Within a Helfrich-Hurault-like picture, this accumulated strain can be accommodated through localized undulation of the cholesteric layers. The BARCODE results support the temporal sequence underlying this interpretation. FCD onset follows the decline of broadly distributed tactoid-scale motion and coincides with a sharp, spatially heterogeneous dynamical event. Across the 8, 30, and 120 J/mL samples at 150 mmol/kg, the magnitude of this late response increases with FCD abundance, while the subsequent decay becomes progressively faster. The combined observations therefore support a kinetic-elastic picture in which rapid formation of an extended layered precursor, progressive restriction of structural rearrangement, and continued evaporative compression create the conditions for the late collective deformation associated with FCD formation.

### Structure and Photonic Response

The preceding sections show that salt and sonication alter both the lateral domain morphology and the internal layered structure. We next examine how these changes affect two distinct optical properties: spectral bandwidth and angular dispersion. Angle-resolved reflection maps were acquired from 200 μm regions and compared with SEM images of the corresponding film cross sections (Figure 7). Spatially averaged UV–vis attenuation spectra and macroscopic film photographs provide complementary information over larger areas (Figure 1 and SI Figure S2). A narrow spectral bandwidth indicates a comparatively restricted range of reflected wavelengths within the selected region, whereas weak angular dispersion indicates that the reflection maximum changes little with viewing angle.

**Figure 7:**
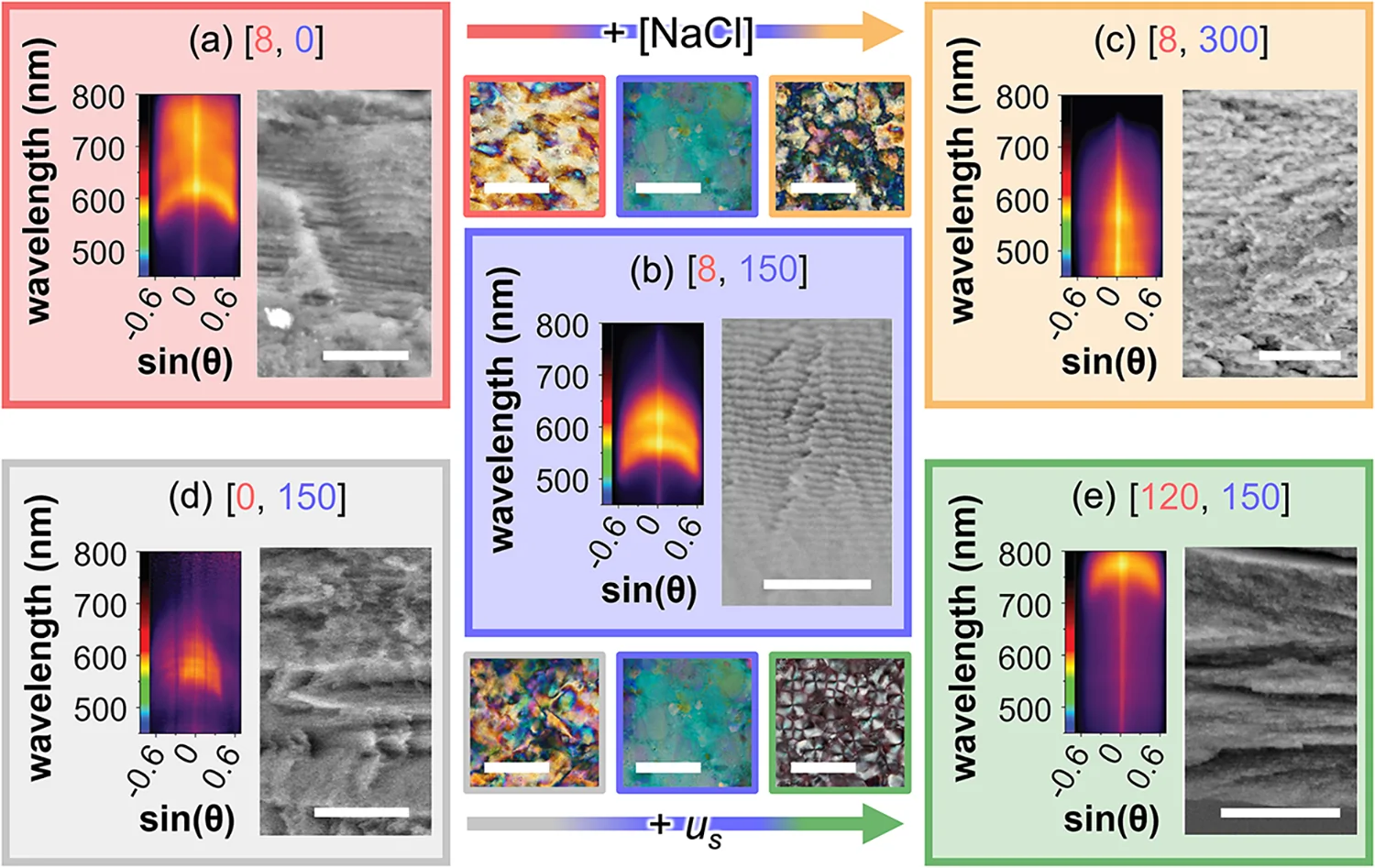
(a–e) Angle-resolved reflectance spectra and scanning electron micrographs of five CNC films. For each film, the left side shows the angle-resolved reflectance spectrum collected from a 200 μm region, with the left color bar denoting the corresponding color of the light wavelength and the right plot denoting the reflectivity normalized to the maximum of each individual color map, ranging from 0 (black) to 100% (yellow). The right side shows an SEM image of an exposed film cross-section (scale bars: 10 μm). The POM images from Figure 2 at the top (corresponding to a, b, c) and bottom (corresponding to b, d, e) depict the film structure as seen through POM (Scale bars: 200 μm), with color-coded outlines corresponding to panels a–e.

For each film, we examined both its photonic response and cholesteric domain morphology. In the angle-resolved maps, bright regions indicate the wavelengths and external angles at which the relative reflected intensity is greatest. Additional maps obtained from 20 and 200 μm circular regions are provided in SI Figure S17. Films [8 J/mL, 0 mmol/kg] and [8 J/mL, 300 mmol/kg] (Figure 7a,c) display broader spectral responses, while [8 J/mL, 150 mmol/kg] and [120 J/mL, 150 mmol/kg] (Figure 7b,e) exhibit narrower local responses. These trends correspond to the SEM observations. Broader spectra occur in films with greater variation in layer orientation and spacing, whereas narrower spectra occur in more coherent domains. Notably, [8 J/mL, 150 mmol/kg] shows a uniform cross-section spanning several hundred micrometers.

Most spectra display a curved angular dependence. This is expected for planar cholesteric films according to eqs 1, 2. Films with large domains and long-range order, such as [8 J/mL, 150 mmol/kg], show a pronounced blueshift of the reflection maximum with increasing external angle (Figure 7b). Films with smaller and more broadly oriented domains show a weaker displacement of the ensemble-averaged maximum. The angular dependence of [8 J/mL, 300 mmol/kg] is strongly reduced over the measured range. Several spectra also exhibit higher-order reflection bands (Figure 7a,b,d), as previously reported in CNC films.^48^

Salt concentration has a strong influence on the photonic response. Samples with either 0 or 300 mmol/kg NaCl show wider local spectral responses, whereas the 150 mmol/kg samples are generally narrower. This trend is consistent with enhanced tactoid coalescence in the intermediate-salt samples. Sonication dose plays a subtler role among the three films at 150 mmol/kg (Figure 7b,d,e). The unsonicated film shows a mismatch between maps collected over small and large regions, consistent with its broad distribution of domain sizes and colors in Figure 2. The UV–vis spectral widths in SI Table S2 provide a complementary spatially averaged measure and show minima at *u*_s_ = 30 J/mL for both 150 and 300 mmol/kg.

To compare the local spectral bandwidth and angular dispersion quantitatively, angle-resolved reflection maps were analyzed for three representative films (SI Figure S16). Wavelength cross sections extracted at the microscope-defined external angles θ = 0° and ± 30° are shown in SI Figure S16b,d,f. Gaussian fits to the spectra at θ = 0° give fwhm values of 57, 43, and 58 nm for the [8 J/mL, 0 mmol/kg], [8 J/mL, 150 mmol/kg], and FCD-rich [120 J/mL, 150 mmol/kg] films, respectively (SI Figure S16b,d,f). The FCD-rich film therefore retains a well-defined local selective-reflection band, although its fitted bandwidth is not the narrowest among the three films.

The mean blue-shift magnitudes between θ = 0° and ±30° are 33, 26, and 26 nm for the [8 J/mL, 0 mmol/kg], [8 J/mL, 150 mmol/kg], and FCD-rich [120 J/mL, 150 mmol/kg] films, respectively. Their reflection maxima at θ = 0° differ substantially, with values of 618, 563, and 774 nm. Because the absolute angular wavelength displacement depends on this initial wavelength scale, the shifts were also compared after normalization by the corresponding central reflection wavelength.

For a locally planar cholesteric domain, using eq 2 together with the normal-incidence relation λ_0_ = *n* *p* gives

3 $$\frac{\lambda \left(\theta \right)}{\lambda_{0}}=\sqrt{1-\frac{n_{\mathrm{air}}^{2}}{{\mathrm{n̅}}^{2}}\sin^{2}\theta }$$

The pitch determines the absolute wavelength scale through λ_0_ = *n* *p*, but it does not contribute explicitly to the normalized angular response. Differences in pitch alone therefore cannot explain differences in the fractional angular shift within the locally planar model. For the experimental comparison, the measured central reflection wavelength λ(0°) was used as the reference value.

Normalization by λ(0°) gives mean fractional blue-shift magnitudes of 5.3, 4.7, and 3.3% for the [8 J/mL, 0 mmol/kg], [8 J/mL, 150 mmol/kg], and FCD-rich [120 J/mL, 150 mmol/kg] films, respectively. The FCD-rich film therefore exhibits the weakest normalized angular dispersion.

The measured angle-resolved maps were compared with the locally planar prediction using the literature-reported range *n* = 1.5–1.6 for dry CNC films^44,48^ (SI Figure S16a,c,e). The pitch used for each calculated curve was set by *p* = λ(0°)/ *n* , giving *p* = 618 nm/n, 563 nm/n, and 774 nm/n for the three films. The FCD-rich film shows the largest reduction in angular dispersion relative to the locally planar prediction. Reproducing its measured fractional shift using the planar model would require an effective value of *n* close to 2. Although *n* was not measured independently and some sample-to-sample variation is possible, such a value lies well above the range reported for dry CNC films.^44,48^ Variation in the average optical index alone is therefore unlikely to account for the measured response. The weaker normalized angular dispersion is consistent with averaging over the spatial distribution of local heli*x*-axis orientations produced by the curved focal conic layers.

This reduced angular dependence is also apparent from the full angle-resolved map. Although the FCD-rich film does not have the narrowest spectrum at θ = 0°, its high-intensity reflection region remains confined to the smallest wavelength range across the measured angles (Figure 7e, Figure S16e). This reduced spectral-angular footprint shows that the range of reflected wavelengths changes less across the measured angular interval, consistent with different local heli*x*-axis orientations contributing to the reflection while limiting the displacement of the ensemble-averaged reflection band.

Overall, the spectral fits and normalized angular analysis distinguish two effects of the domain morphology. Locally extended cholesteric regions with a comparatively uniform heli*x*-axis orientation can produce a narrow reflection band while retaining a pronounced angular response. The curved focal conic morphology is consistent with a broader distribution of local heli*x*-axis orientations that contributes to a reduced displacement of the ensemble-averaged reflection maximum while preserving the local periodic cholesteric structure responsible for selective reflection. Among the films examined quantitatively, the FCD-rich film combines a well-defined local reflection band, the smallest spectral-angular footprint, and the weakest normalized angular dispersion.

## Conclusion

In this work, we investigated how salt concentration and sonication dose jointly regulate the self-assembly and photonic response of dish-cast CNC films. Across a matrix of 24 processing conditions, increasing sonication dose red-shifts the cholesteric reflection most strongly at low salt concentration, whereas increasing [NaCl] promotes a blue-shift and progressively reduces the influence of sonication. These processing parameters also alter the drying pathway by changing the CNC particle and bundle population and the kinetics of tactoid nucleation, growth, transport, and coalescence.

The initial parameter grid identified [120 J/mL, 150 mmol/kg] as an FCD-rich condition, while subsequent scans showed that FCD formation extends over a finite processing window. At 150 mmol/kg, FCDs are sparse at *u*_s_ = 8 J/mL, become more frequent at 30 J/mL, and are prominent at 120 J/mL. At fixed *u*_s_ = 120 J/mL, FCDs are absent at 0–50 mmol/kg and appear from 75 to 150 mmol/kg. FCD formation corresponds to a region in which salt concentration and sonication jointly establish a suitable self-assembly pathway.

Time-lapse imaging and analysis identify this pathway quantitatively. At fixed 150 mmol/kg, increasing sonication produces a larger tactoid-associated island population over a progressively shorter interval. For the FCD-rich [120 J/mL, 150 mmol/kg] condition, the number of islands subsequently decreases while the mean island area increases, demonstrating growth and coalescence into fewer, larger cholesteric regions. Mean speed is high during this coalescence stage and begins to decline before FCD formation. The first appearance of Maltese-cross textures is then accompanied by a sharp increase in the spatial heterogeneity of the motion, marking a transition from broadly distributed tactoid-scale motion to strongly localized structural rearrangement.

This late dynamical event becomes increasingly pronounced as FCD abundance increases from 8 to 30 to 120 J/mL, while the subsequent reduction in the speed heterogeneity becomes progressively faster. In contrast, the spatial heterogeneity of the initial nucleation and growth dynamics decreases with increasing sonication. FCD formation therefore emerges as a distinct late-stage transformation, occurring after an extended cholesteric precursor has formed and large-scale structural mobility has begun to decline.

The control experiments further establish FCD formation as a kinetic pathway governed by the coordinated timing of multiple structural and dynamic processes. The unsonicated [0 J/mL, 150 mmol/kg] sample develops a substantial tactoid population and these tactoids coalesce into significantly larger tactoids, similar to FCD-forming samples, but this coalescence and growth is slower and more prolonged, leading to a non-FCD morphology. Conditions at extreme salt or sonication likewise generate strong structural and dynamic responses but enter the birefringent regime substantially earlier in drying and terminate in morphologies without prominent FCDs. Most directly, slowing the evaporation of [120 J/mL, 150 mmol/kg] produces a larger population of persistent small tactoids, gradual coalescence, and lower-amplitude structural dynamics distributed over a much longer interval, and the sharp late FCD-associated event is strongly suppressed. The resulting film is dominated by incompletely coalesced tactoids and domain walls. Together, these controls point toward the kinetic conditions necessary for abundant FCD formation.

The rheological measurements provide complementary evidence that the suspension response evolves with CNC concentration, sonication, and residence time. For the FCD-forming [120 J/mL, 150 mmol/kg] suspension, pronounced shear-sensitive behavior develops at higher CNC concentrations with aging, indicating substantial changes in mesoscale organization. The unsonicated [0 J/mL, 150 mmol/kg] suspension exhibits a different concentration-dependent rheological response and contains a larger characteristic particle and bundle population, accompanied by slower tactoid growth and coalescence and a spatially heterogeneous final film. Because the aged flow curves reflect structural evolution during residence at fixed concentration, they cannot be mapped directly onto the instantaneous rheology of the drying film. They nevertheless demonstrate that the capacity for structural rearrangement changes strongly with concentration and microstructural state, consistent with the distinct tactoid-assembly kinetics observed by BARCODE.

The combined results support a kinetic-elastic mechanism for focal-conic formation. Rapid tactoid generation, growth, and coalescence first produce an extended cholesteric precursor. Continued evaporation then increases CNC concentration, contracts the preferred cholesteric pitch, and reduces film thickness. As structural mobility decreases, the layered structure becomes progressively less able to accommodate these changes by continuous rearrangement. Elastic compression can therefore accumulate until the layers undergo a localized Helfrich-Hurault-like undulation, producing FCDs. The BARCODE analysis directly establishes the temporal separation between precursor assembly and this late localized transformation, while the evaporation-rate control demonstrates that access to the instability depends on the competition between flow conditions and structural relaxation. A late change in the phase-transition pathway, including the spinodal-like ordering proposed previously for residual isotropic CNCs,^47^ could contribute to formation of the coherent precursor. However, the present measurements do not uniquely distinguish this process from rapid nucleation and tactoid coalescence.

The resulting domain morphology strongly influences the optical response. Locally extended and coherent cholesteric regions can exhibit narrow spectral responses while retaining pronounced angular dispersion. In contrast, the FCD-rich [120 J/mL, 150 mmol/kg] film combines a well-defined local reflection band with weak normalized angular dispersion. The curved layers within the FCDs introduce a distribution of local heli*x*-axis orientations that reduces the angular dependence of the reflected color while preserving the periodic cholesteric structure responsible for selective reflection.

In conclusion, salt concentration and sonication dose provide coupled control over cholesteric pitch, self-assembly kinetics, domain morphology, spectral bandwidth, and angular color response. More broadly, these results show that the optical properties of CNC films can be tuned not only through equilibrium structural parameters, but through control of the pathway by which ordered layers nucleate, grow, coalesce, deform, and become retained during evaporation. Further manipulation of confinement geometry and surface anchoring may provide additional control over FCD size, density, and orientation. Deliberate control of these self-assembled defects could provide a general strategy for introducing controlled structural disorder into CNC photonic films, allowing spectral selectivity and iridescence to be engineered through defect organization.

## Methods

### CNC Synthesis

Cellulose nanocrystals were obtained from the acid hydrolysis of Whatman no.1 cellulose filter paper (35 g). The cellulose source was shredded with a spice grinder (Waring WSG60K). We prepared 490 mL of 64 wt % sulfuric acid (Sigma-Aldrich, 95–98%) and heated it to 66 °C. The filter paper was added to the acid solution, stirred vigorously with a mechanical stirrer for 30 min during hydrolysis, and the reaction was quenched with five liters of deionized (DI) water. The resulting suspension was allowed to sediment overnight, and the clear supernatant was decanted. The sedimented material was centrifuged in 20 min cycles at 15,000 RCF until it was all in pellet form. This was followed by additional centrifugation cycles, consisting in the addition of DI water until the CNCs in the supernatant were suspended. Only the supernatant was collected and the pellet was discarded. This suspension was dialyzed over 2 weeks in DI water (replenished daily) with MWCO 12–14 kDa dialysis membranes (Spectrum Spectra/Por 4). This procedure yielded a 1.1 wt % CNC suspension with a total volume of around 900 mL.

### Elemental Analysis and Nominal Sulfate Content

The total sulfur content of freeze-dried CNCs was measured by inductively coupled plasma optical emission spectroscopy (ICP-OES) using a PerkinElmer Optima 8300 instrument. The retained analytical summary reported a sulfur mass fraction of 0.71 wt % relative to the CNC dry mass, corresponding to 221 mmol S kg^–1^ CNC. Assuming one sulfur atom per sulfate half-ester and that the measured sulfur originates from sulfate half-esters introduced during sulfuric-acid hydrolysis, the result represents a nominal sulfate half-ester content of 221 mmol kg^–1^ CNC. Using the relation described by Hamad and Hu,^49^ the measured sulfur mass fraction corresponds to approximately 3.7 sulfate groups per 100 anhydroglucose units. ICP-OES quantifies total sulfur and does not independently determine the fraction of sulfate groups that is surface-accessible or ionized.

### Preparation of CNC Suspensions

The stock CNC suspension was divided and diluted into six separate suspensions (25 mL, [CNC] = 3.5 wt %) in 50 mL centrifuge tubes (Corning Falcon). These suspensions were tip sonicated using a Hielscher UP200 St ultrasonic processor with a 7 mm titanium tip for various time intervals (corresponding to 0 to 1440 J per mL of CNC suspension) under the following conditions: 30 W, 70:30 ON:OFF cycles, with the tip submerged halfway into the sample. The suspensions were immersed in an ice bath during sonication to prevent sample heating and potential desulfation. For sonication times longer than 5 min, 10 min pauses every 5 min were taken to replenish the ice bath and cool the sample. After sonication, each of the six suspensions was divided and diluted (with DI water and a 0.2 M aqueous NaCl solution) into four separate suspensions ([CNC] = 3.0 wt %) with different NaCl concentrations (0 to 450 mmol of NaCl per kilogram of CNC) making a total of 24 CNC samples. These CNC suspensions (or films) that underwent a combination of *u*_s_ and/or [NaCl] will be noted as [“*x*” J/mL, “*y*” mmol/kg] or [“*x*”, “*y*”]. For example, [120, 150] or [120 J/mL, 150 mmol/kg] refers to a CNC film with *u*_s_ = 120 J/mL and [NaCl] = 150 mmol/kg.

### CNC Film Formation

The films presented in this work were evaporated at an initial CNC concentration of 3 wt %, in the isotropic phase. This is necessary to have every suspension start the evaporation process equally in the isotropic phase. Each of the 24 CNC suspensions was dropcast (3 mL, 3 wt %) into a glass Petri dish (3.5 cm in diameter, DURAN Steriplan). All CNC suspensions were evaporated simultaneously at around 50–60% RH and 20 °C. The evaporation process usually took 48 to 60 h to complete.

### Polarized Optical Microscopy

The center of the CNC films (within a 1 cm diameter circle) was imaged in transmission between crossed polarizers on a Nikon Ti-E inverted microscope equipped with a T-P2 DIC Polarizer module and an analyzer. The images and videos were captured with a Nikon DS-Ri2 CMOS camera. The time-lapse videos of evaporating CNC suspensions were recorded at 1 frame per minute. The videos were captured under software-side light autoexposure, varying from 1 s at evaporation start to around 30 ms on evaporation completion. This was done to ensure adequate light exposure when recording any birefringence, at the cost of overexposing frames with no birefringence.

### Optical Microscopy

The center of the colored films was imaged in reflection, with a left circular polarizer in the light path, on a Leica DM IL LED inverted microscope. The photographs were taken with a mounted Nikon Z6 digital camera.

### UV–Vis Transmission Spectrometry

UV–vis transmission spectra for the center of the films were obtained using a LAMBDA 365 UV/vis spectrophotometer. For all spectra, the acquisition rate was 960 nm/min, in a wavelength range of 400 to 1100 nm. For each film, a rectangular region of about 1 cm by 0.5 cm was analyzed in its center.

### Scanning Electron Microscopy (SEM)

The morphological properties of CNC films were observed using a Thermo Scientific Phenom XL G2 Desktop scanning electron microscope at 15 kV and 60 Pa, with a working distance of 6 to 7 mm. To observe the inner structure of the CNC films, we submerged them in liquid nitrogen for 1 min and then fractured them with tweezers to expose the cross section.

### Viscosity Measurement

The rheological measurements were performed with a rotational rheometer (MCR300, Anton Paar), using cone–plate geometry. The cone had a diameter of 50 mm, a cone angle of 1.001°, and a truncation of 0.051 mm. For all measurements, the temperature was set at 25 °C and 1 mL of sample was applied before trimming to ensure complete coverage. Each sample was analyzed over a shear-rate interval of 0.1–150 s^–1^ using 50 data points over 5 min. Solvent evaporation was minimized during the measurement. For the data set in Figure S13, samples were concentrated to 10 wt % at room temperature after 1 week. The 10 wt % suspensions were divided and diluted to prepare the 4, 6, and 8 wt % samples. Measurements were performed 3 days after. The measurements were subsequently repeated using freshly prepared and one-month-aged suspensions at 4, 6, 8, and 10 wt % CNC, where aging time is the residence time at a target CNC concentration.

### Time-Resolved Image Analysis

Each polarized optical microscopy (POM) time-lapse was acquired as a stack of 1608 × 1608-pixel TIFF images. Images were converted from RGB to 16-bit grayscale using Fiji/ImageJ and divided into four 804 × 804-pixel quadrants, corresponding to 563 × 563 μm^2^, using a Python script. Each quadrant was analyzed independently using the BARCODE software.^45,46^ The conditions analyzed were [0 J/mL, 150 mmol/kg], [0 J/mL, 450 mmol/kg], [8 J/mL, 150 mmol/kg], [30 J/mL, 150 mmol/kg], [120 J/mL, 150 mmol/kg], [1440 J/mL, 0 mmol/kg], and the [120 J/mL, 150 mmol/kg] slow-evaporation condition. BARCODE converts each grayscale image into a binarized representation and calculates an optical-flow field from successive analyzed frames (see Figure 6a). For the comparisons shown in SI Figures S9, S11, and S12, images were analyzed at 10-frame intervals.

From the binarized images, BARCODE identifies connected regions of white pixels, referred to as “islands.” The Number of Islands gives the number of disconnected segmented regions within the field of view (FOV), while the Mean Island Area gives the average projected area of these regions as a percentage of the FOV. During tactoid nucleation and growth, these quantities provide measures of the population and characteristic size of disconnected birefringent regions. Once FCDs form, the Number of Islands no longer corresponds directly to the number of physical domains because a single Maltese-cross texture can be segmented into multiple islands. Binarization thresholds were selected separately for each time-lapse to suppress spurious detections before cholesteric nucleation while retaining sensitivity to the earliest persistent birefringent tactoids.

Optical-flow analysis quantifies frame-to-frame changes in the POM texture. The Mean Speed is calculated as the spatial mean of the magnitudes of all optical-flow vectors within the FOV and therefore reports the overall magnitude of image-texture evolution, including contributions from tactoid translation, growth, deformation, and coalescence. The Speed Standard Deviation (Speed SD) is calculated from the same distribution of vector magnitudes and reports the spatial heterogeneity of this activity. A relatively small Speed SD corresponds to motion of more similar magnitude throughout the FOV, whereas a larger Speed SD indicates stronger spatial variation between more and less dynamically active regions. For comparisons across each image, values from the four quadrants were averaged, with the corresponding interquadrant variability shown in the BARCODE plots.

### Angle-Resolved Spectroscopy

Angle-resolved reflectivity spectra were acquired using a home-built optical setup based on a Nikon Ti–U inverted microscope. Broadband illumination (450–800 nm) from a Nikon halogen lamp was directed to the sample through a 50/50 beamsplitter and focused using a 60× air objective (Nikon ELWD, CFI Plan Fluor, NA = 0.7). The sample was therefore illuminated simultaneously over the full external angular range defined by the objective, approximately ±44.4° in air. Reflected light was collected by the same objective and relayed to an Andor Kymera 193i imaging spectrometer equipped with a 150 lines mm^–1^ reflective grating and an Andor iXon Ultra 888 electron-multiplying CCD detector. A Fourier lens positioned one focal length behind the collimation lens imaged the objective back focal plane onto the spectrometer entrance plane, mapping each reflection angle to a distinct spatial position. A slit at the spectrometer entrance selected a one-dimensional slice of the back focal plane, and the light was dispersed perpendicular to the angular axis. Circular sample regions with diameters of 20 or 200 μm were selected by adjusting an aperture in the first image plane outside the microscope. Raw angular reflectivity maps were divided by a reference measurement obtained from a flat silver mirror to correct for the spectral profile of the lamp and the wavelength-dependent optical throughput. Each displayed map was subsequently normalized to its own maximum intensity. The maps therefore share a common relative 0–1 display scale but do not preserve absolute reflectivity differences between samples.

## Supplementary Material



















## Notes

Complete grid of reflection-mode optical micrographs for the CNC film parameter space (Figure S1) and macroscopic photographs of structurally colored films (Figure S2); analysis of the nominal CNC sulfate half-ester content and comparative conductivity measurements of selected suspensions (SI Table S1); atomic force microscopy (AFM) and dynamic light scattering (DLS) characterization of the sonication-dependent CNC particle and bundle population (Figures S3 and S4), together with spatially averaged UV–vis spectral widths (SI Table S2); refined scans of the FCD-forming processing window at fixed sonication dose (Figure S5) and across salt concentration and sonication dose (Figure S6), as well as reproduction of FCD formation across independent CNC synthesis batches (Figure S7); the slow-evaporation control for the FCD-forming [120 J/mL, 150 mmol/kg] condition (Figure S8); quantitative BARCODE analysis of the self-assembly dynamics, including absolute-time trajectories at fixed ionic content (Figure S9), event-aligned comparisons of tactoid nucleation, growth, and FCD formation (Figure S10), comparison with extreme salt and sonication conditions (Figure S11), and the effect of evaporation rate at fixed composition (Figure S12); concentration- and residence-time-dependent rheology (Figure S13) and empirical power-law analysis of the 8 wt % CNC concentration-series flow curves (Figure S14 and SI Table S3); a filtration control examining the effect of large aggregates on dried-film morphology (Figure S15); a density-contrast measurement and discussion of buoyancy-associated convection; quantitative analysis of local spectral bandwidth and normalized angular dispersion for three representative films (Figure S16 and SI Table S4); complete angle-resolved reflection maps collected from 20 and 200 μm regions together with the corresponding SEM cross sections (Figure S17) (PDF)
Evaporation time-lapse of sample [120 J/mL, 150 mmol/kg] (MP4)
Evaporation time-lapse of sample [120 J/mL, 150 mmol/kg] at a slowed evaporation rate. The video starts at the 188-hour mark (still in the isotropic regime) (MP4)
Evaporation time-lapse of sample [0 J/mL, 150 mmol/kg] (MP4)
Evaporation time-lapse of sample [8 J/mL, 150 mmol/kg] (MP4)
Evaporation time-lapse of sample [30 J/mL, 150 mmol/kg] (MP4)
Evaporation time-lapse of sample [0 J/mL, 0 mmol/kg] (MP4)
Evaporation time-lapse of sample [1440 J/mL, 0 mmol/kg] (MP4)
Evaporation time-lapse of sample [0 J/mL, 450 mmol/kg] (MP4)
