# Supplementary material for: Kinetic Access to Focal Conic Domains in Photonic Cellulose Nanocrystal Films
Source: ACS Nano. 2026 Oct 23;20(44):30350–65. doi: 10.1021/acsnano.6c18491 (PMC13758399; doi:10.1021/acsnano.6c18491)
Supplement: Supplementary file 1 [file nn6c18491_si_001.pdf]

Supporting Information:

Kinetic access to focal conic domains in photonic cellulose nanocrystal films

Diogo V. Saraiva,[†] Lotte Polling,[†] Sander J. W. Vonk,[†,¶] Samaneh Rahbar,[‡] Ivo R. Vermaire,[†] Djouke A. C. Elferink,[†] Rae M. Robertson-Anderson,[‡] Freddy T. Rabouw,[†] and Lisa Tran*,[†]

†*Debye Institute for Nanomaterials Science, Utrecht University, 3584 CC Utrecht, The Netherlands*

‡*Department of Physics and Biophysics, University of San Diego, San Diego, CA 92110, United States*

¶*Optical Materials Engineering Laboratory, Department of Mechanical and Process Engineering, ETH Zurich, Zurich 8092, Switzerland*

E-mail: l.tran@uu.nl

The Supporting Information includes a complete grid of reflection-mode optical micrographs for the CNC film parameter space (Figure S1) and macroscopic photographs of structurally colored films (Figure S2); analysis of the nominal CNC sulfate half-ester content and comparative conductivity measurements of selected suspensions (SI Table S1); atomic force microscopy (AFM) and dynamic light scattering (DLS) characterization of the sonication-dependent CNC particle and bundle population (Figs. S3 and S4), together with spatially averaged UV-Vis spectral widths (SI Table S2); refined scans of the FCD-forming processing window at fixed sonication dose (Figure S5) and across salt concentration and sonication dose (Figure S6), as well as reproduction of FCD formation across independent CNC synthesis batches (Figure S7); the slow-evaporation control for the FCD-forming [120 J/mL, 150 mmol/kg] condition (Figure S8); quantitative BARCODE analysis of the self-assembly dynamics, including absolute-time trajectories at fixed ionic content (Figure S9), event-aligned comparisons of tactoid nucleation, growth, and FCD formation (Figure S10), comparison with extreme salt and sonication conditions (Figure S11), and the effect of evaporation rate at fixed composition (Figure S12); concentration- and residence-time-dependent rheology (Figure S13) and empirical power-law analysis of the 8 wt.% CNC concentration-series flow curves (Figure S14 and SI Table S3); a filtration control examining the effect of large aggregates on dried-film morphology (Figure S15); a density-contrast measurement and discussion of buoyancy-associated convection; quantitative analysis of local spectral bandwidth and normalized angular dispersion for three representative films (Figure S16 and SI Table S4); complete angle-resolved reflection maps collected from 20 and 200 µm regions together with the corresponding SEM cross-sections (Figure S17); and polarized optical microscopy time-lapse videos of the evaporation and self-assembly dynamics for eight representative conditions (SI Videos 1-8).

Complete grid with LCP reflection images

Reflection images (through a left circular polarizer) were captured for each CNC film. This showcases all 24 CNC films, including the ones that do not exhibit any visible structural color.

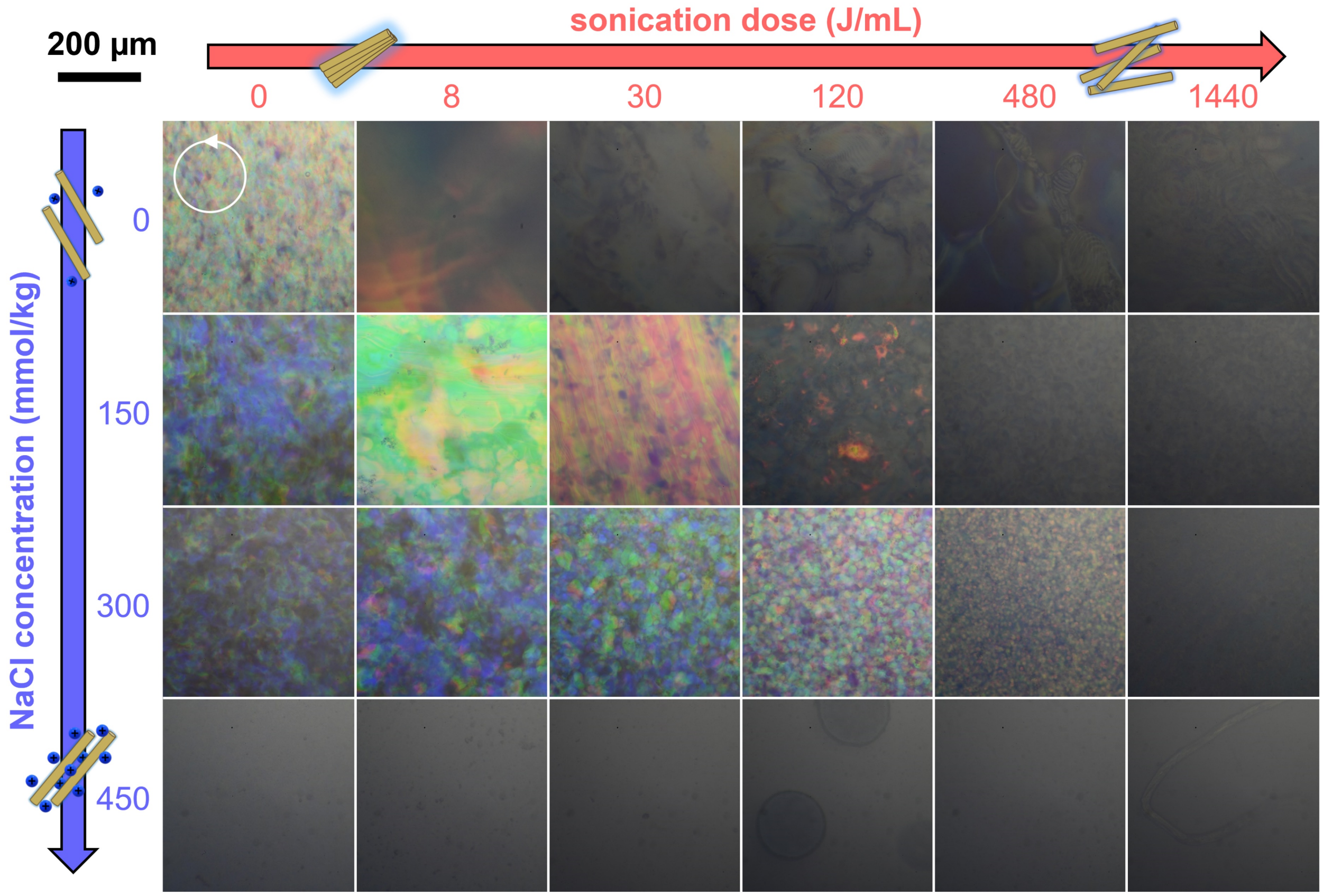

Figure S1: An array of microscope images taken in reflection mode, captured through a left circular polarizer. All pictures were taken within a 5 mm region around the center of the film.

Photographs of CNC films

Macroscopic photographs of a selection of films were captured with the camera of a Samsung Galaxy S21 FE. The films were illuminated with a white ring light behind the camera and photographed at a 12° angle with the substrate. In SI Figure S2, we show most of the CNC films that display visible structural color at a macroscopic scale.

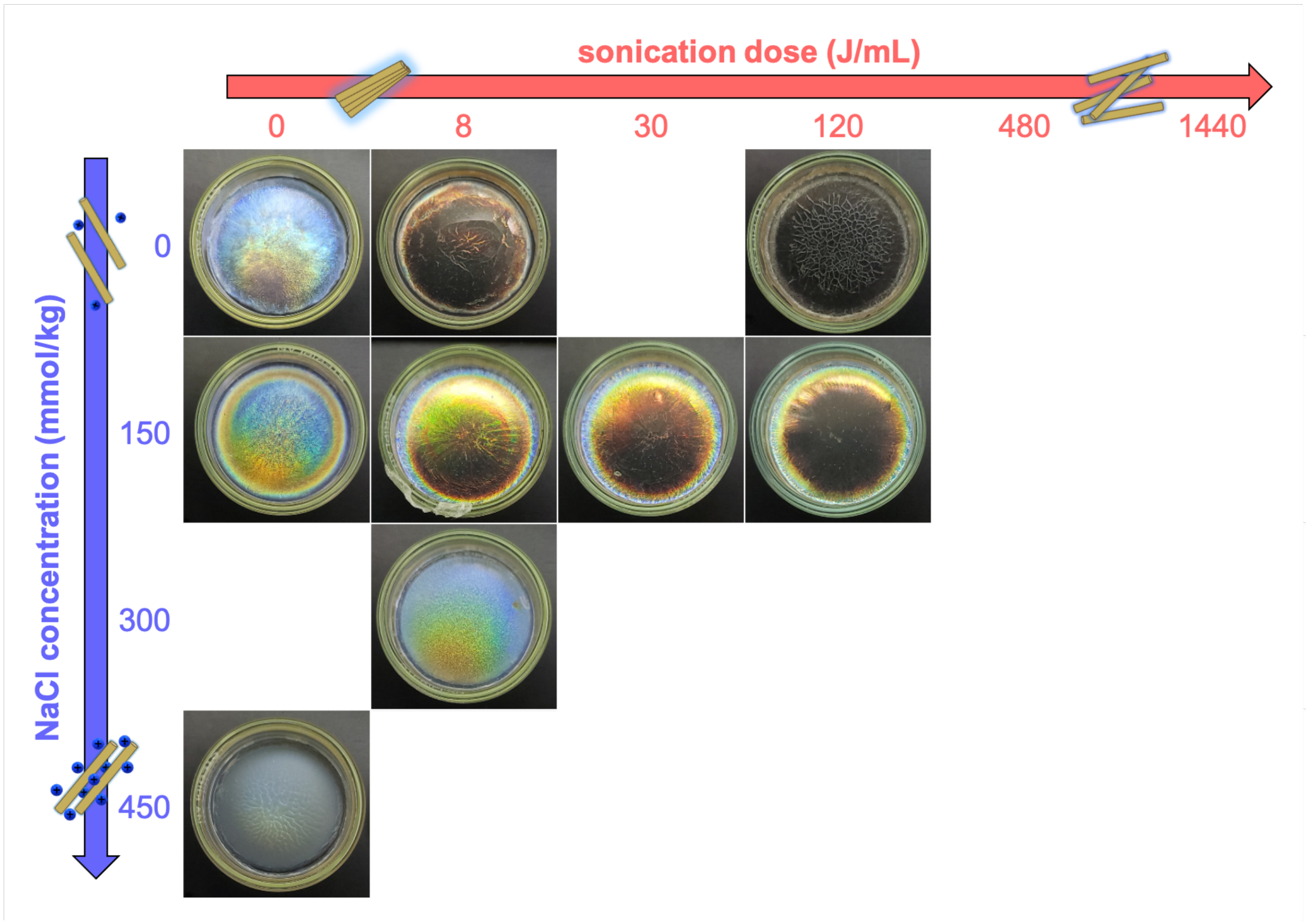

Figure S2: An array of photographs of CNC films presented in this work. For scale, the diameter of all petri dishes is 3.5 cm.

The majority of the films exhibit a coffee-ring morphology, characterized by increased material accumulation near the dish perimeter and a corresponding spatial variation in structural color. This effect is mostly reported in evaporated sessile droplets,[S1–S3] but is also present in films.[S4]

Estimation of nominal CNC sulfate content

The sulfur content of freeze-dried CNCs was quantified by inductively coupled plasma-atomic emission spectroscopy (ICP-OES) using a PerkinElmer Optima 8300 instrument. Samples (≈50 mg of solid CNC material) were digested in concentrated nitric acid (65%) at 120 °C until complete dissolution. The digests were diluted to a defined volume with ultrapure water before analysis. The measurement is used here to estimate the nominal sulfate half-ester content.

A sulfur mass fraction of 0.71 wt.% corresponds to 7.1 g S per kg of dry CNC. The corresponding molar sulfur content is

$$c_S = \frac{7.1 \text{ g S kg}^{-1} \text{ CNC}}{32.065 \text{ g mol}^{-1}} = 0.221 \text{ mol S kg}^{-1} \text{ CNC}, \tag{1}$$

or 221 mmol S kg^{-1} CNC. Assuming that the measured sulfur originates from sulfate half-esters introduced during sulfuric-acid hydrolysis and that each sulfate half-ester contains one sulfur atom, this value corresponds to a nominal sulfate half-ester content of 221 mmol kg^{-1} CNC. ICP-OES measures total sulfur and does not independently determine whether the sulfur-containing groups are located at the CNC surface, accessible to the surrounding solution, or ionized under the suspension conditions.

The number of sulfate groups per 100 anhydroglucose units was estimated using the approach described by Hamad and Hu:[S5]

$$N_{\text{sulfate}} = \frac{100 M_{\text{AGU}} w_S}{M_S - M_{\text{SO}_3} w_S}, \tag{2}$$

where $M_{\text{AGU}} = 162.14$ g mol^{-1} is the molar mass of an anhydroglucose unit, $M_S = 32.065$ g mol^{-1} is the molar mass of sulfur, $M_{\text{SO}_3} = 80.06$ g mol^{-1} is the mass added to an anhydroglucose unit upon formation of a sulfate half-ester, and $w_S = 0.0071$ is the measured sulfur mass fraction. Substitution gives

$$N_{\text{sulfate}} = \frac{100(162.14)(0.0071)}{32.065 - (80.06)(0.0071)} = 3.65, \tag{3}$$

corresponding to approximately 3.7 sulfate groups per 100 anhydroglucose units. This result is a nominal sulfate half-ester density calculated from the retained total-sulfur value and the assumptions stated above.

Ionic content and conductivity measurements

The sulfate half-ester groups introduced during sulfuric-acid hydrolysis give the CNCs a negative surface charge and are accompanied by counterions in the aqueous suspension. These CNC-associated ions contribute to the ionic environment even before NaCl is added. The nominal sulfate half-ester density of 221 mmol kg^{-1} CNC corresponds to approximately 6.6 mmol L^{-1} sulfate charge equivalents at 3 wt.% CNC, although the fraction of the associated counterions that remains freely mobile is not known. Added NaCl introduces additional sodium and chloride ions and increases electrostatic screening between the CNCs.

Because the complete mobile-ion population and the extent of counterion association are not known, the ionic strength and Debye length cannot be determined reliably from the nominal NaCl concentration alone. Electrical conductivity was therefore measured as an experimental comparison of the mobile ionic environments of selected suspensions. Conductivity is not equivalent to ionic strength because it also depends on ion identity, mobility, valence, and association, but measurements performed under the same conditions allow the relative effects of added salt and sonication to be compared.

Tip sonication has previously been associated with modest increases in the conductivity of aqueous CNC suspensions, possibly because sonication changes the association and mobility of CNC counterions.[S6,S7] To compare this effect with that of added NaCl, conductivity measurements were performed on 7 mL CNC suspensions under ambient conditions using an Eutech CON 450 conductivity meter. All measurements were conducted at 3.5 wt.% CNC. The results are summarized in SI Table S1.

Table S1: Electrical conductivity of selected CNC suspensions measured at 3.5 wt.% CNC. Salt concentrations are reported in mmol NaCl/kg CNC.

**Sample**	**Conductivity (mS/cm)**
[0 J/mL, 0 mmol/kg]	1.20
[8 J/mL, 0 mmol/kg]	1.22
[1440 J/mL, 0 mmol/kg]	1.41
[0 J/mL, 150 mmol/kg]	3.28
[8 J/mL, 150 mmol/kg]	3.20
[30 J/mL, 150 mmol/kg]	3.25
[120 J/mL, 150 mmol/kg]	3.31
[8 J/mL, 300 mmol/kg]	4.26

Without added NaCl, the conductivity is approximately 1.20 mS/cm for the unsonicated suspension and 1.22 mS/cm after sonication at 8 J/mL. Increasing the sonication dose to 1440 J/mL raises the conductivity to 1.41 mS/cm. Sonication can therefore produce a measurable increase in conductivity in the absence of added salt, although the magnitude of this change remains modest over the conditions examined.

Adding 150 mmol NaCl/kg CNC increases the conductivity to approximately 3.2-3.3 mS/cm. Within this salt series, the conductivity varies only from 3.20 to 3.31 mS/cm as the sonication dose increases from 0 to 120 J/mL, and no systematic sonication-dependent trend is resolved. Increasing [NaCl] to 300 mmol/kg further raises the conductivity to 4.26 mS/cm. Thus, within the salt and sonication range associated with FCD formation, added NaCl changes the measured conductivity much more strongly than sonication.

These measurements support a qualitative interpretation in which added NaCl is the dominant control over the mobile ionic environment, whereas sonication primarily changes the CNC particle

and bundle population over the moderate sonication range examined. The conductivity data do not exclude smaller changes in counterion association or ion mobility, nor do they provide a direct measure of the electrostatic screening length. They are therefore used to qualitatively compare the prepared suspensions.

The measurements were performed at 3.5 wt.% CNC, near the initial concentration used for film preparation, and are not extrapolated quantitatively through evaporation. During drying, the concentrations of CNCs and mobile ions increase together, while counterion association and ion mobility may also change. Consequently, the values in SI Table S1 characterize the starting suspensions and not the evolving ionic environment of the drying films.

Effect of sonication on CNC particle and bundle size

Atomic force microscopy (AFM) and dynamic light scattering (DLS) were used to determine whether increasing sonication dose changes the characteristic size distribution of the CNC population. The two methods provide complementary information. AFM measures the projected dimensions of individual objects deposited on a substrate, whereas DLS reports an apparent hydrodynamic size for particles and aggregates dispersed in liquid. Because the CNCs are anisotropic and polydisperse, the values obtained using the two methods are not expected to be quantitatively interchangeable. They are instead used together to assess the qualitative change in particle and bundle size with sonication dose.

Atomic force microscopy

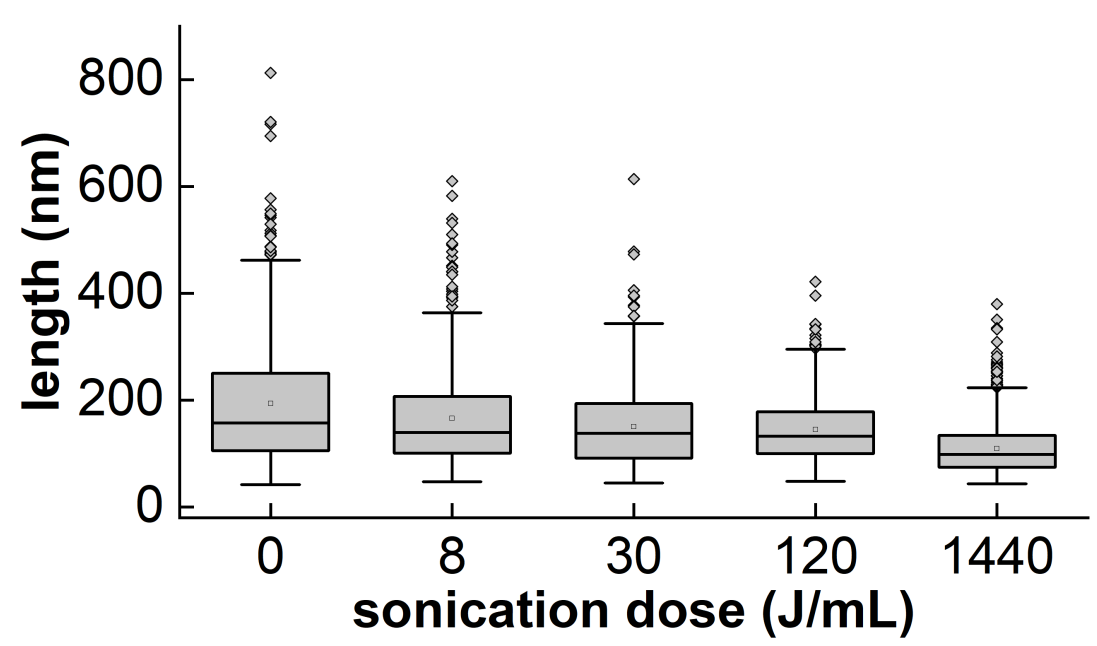

Figure S3: Projected length distributions of CNC objects measured by AFM after sonication at $u_s = 0$, 8, 30, 120, and 1440 J/mL. A total of 600 objects were measured for each sonication dose. The boxes represent the interquartile range, the horizontal lines indicate the median, and the square markers denote the mean. The whiskers extend to 1.5 times the interquartile range, while the lower and upper crosses indicate the 1st and 99th percentiles, respectively. The distributions shift toward shorter projected lengths with increasing sonication dose.

A freshly cleaved mica sheet (Ted Pella, V1 Mica, 25 × 25 mm) was used as the AFM substrate. The mica surface was functionalized by applying 20 µL of 0.01 wt.% poly-L-lysine for 45 s, followed by thorough rinsing with ultrapure water and drying under nitrogen. Subsequently, 200 µL of a 0.004 wt.% CNC suspension was applied to the substrate and allowed to adsorb for 45 s. The surface was then rinsed with ultrapure water and dried under nitrogen. Samples were prepared from CNC suspensions exposed to sonication doses of 0, 8, 30, 120, and 1440 J/mL.

AFM images were acquired using a NanoWizard ULTRA Speed 2 instrument (Bruker) with FASTSCAN-A probes operated in tapping mode. The probes had a nominal resonance frequency of 1.4 MHz and spring constant of 17 N/m. For each sonication dose, the projected lengths of 600 deposited CNC objects were measured using Gwyddion version 2.62. The resulting distributions are shown in SI Figure S3.

The AFM distributions show a progressive reduction in the projected length of the deposited objects with increasing sonication dose. The unsonicated sample contains a larger population of long objects, which may include individual long CNCs, laterally associated particles, and CNC bundles. Increasing u_s reduces the occurrence of these long objects and shifts the distribution toward shorter lengths. AFM imaging alone cannot determine whether this change results exclusively from separation of bundles, shortening of individual crystallites, or a combination of both processes. The

results nevertheless demonstrate that sonication substantially changes the characteristic dimensions of the deposited CNC population.

Dynamic light scattering

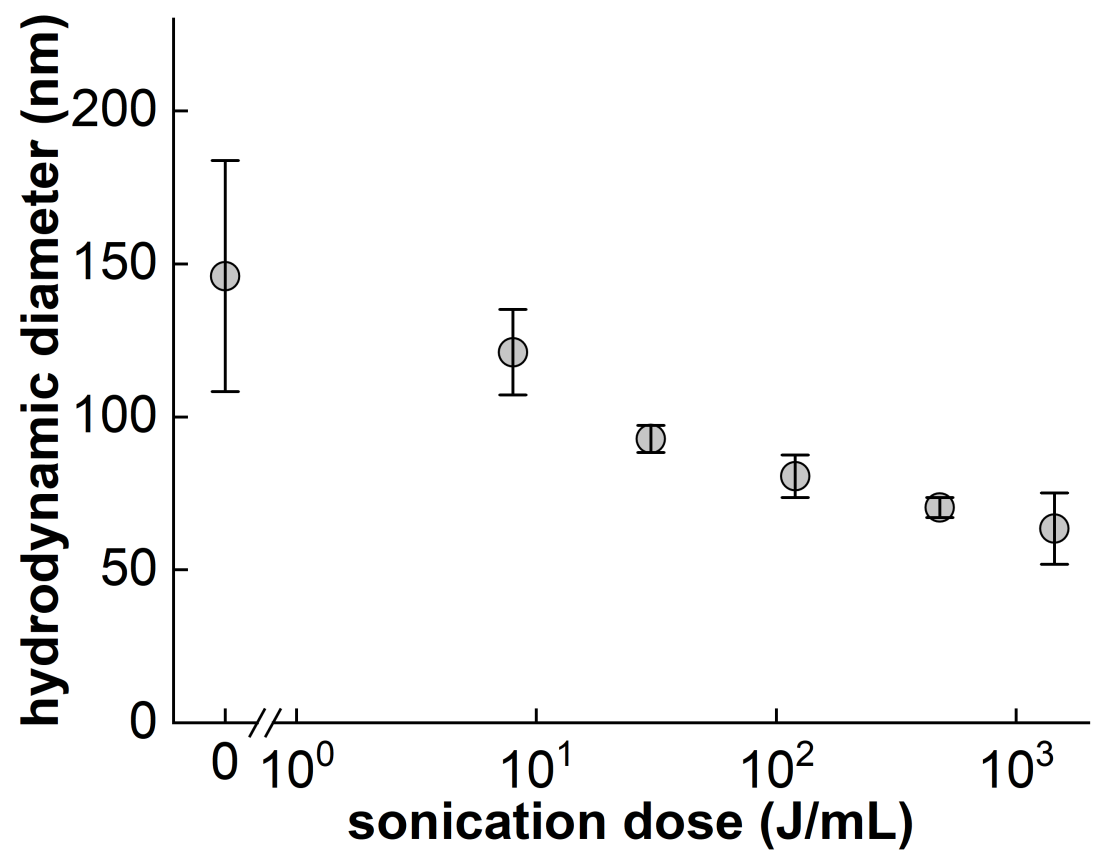

Figure S4: Apparent hydrodynamic size of dilute CNC suspensions measured by DLS as a function of sonication dose. The vertical axis represents the z-average hydrodynamic diameter as reported by the DLS software. The DLS-derived size decreases with increasing sonication dose, consistent with disruption of larger CNC objects and bundles. The error bars represent the standard deviation across three measurement cycles of 20 runs.

The apparent hydrodynamic diameter of the CNC population was also measured by DLS. Measurements were performed at a CNC concentration of 0.15 wt.% using DTS1070 capillary cells and a Malvern Zetasizer Nano instrument. For each sonication dose, three measurement cycles of 20 runs were performed.

DLS similarly shows a decrease in apparent hydrodynamic size with increasing sonication dose. Because the scattered-light intensity is strongly weighted toward larger objects, DLS is particularly sensitive to residual aggregates and bundles. The decrease in the DLS-derived size therefore supports a reduction in the population of large dispersed objects after sonication. Quantitative comparison with the AFM lengths is not appropriate because DLS represents anisotropic CNCs using an effective hydrodynamic measure, while AFM measures dried and surface-deposited objects.

Combined interpretation

Together, the AFM and DLS measurements establish that sonication reduces the characteristic size of the CNC particle and bundle population. The measurements support disruption of larger bundles, although they do not distinguish bundle separation from possible shortening of individual CNCs at the highest sonication doses. These size-distribution changes provide experimental support for the interpretation that sonication modifies the number density, effective interaction volume, and chiral contribution of the dispersed CNC population. They also provide structural context for the rheological and film-morphology results discussed below. In particular, the presence of larger objects in the unsonicated population is consistent with the heterogeneous flow response of [0 J/mL, 150 mmol/kg] and with the improved film uniformity obtained after filtration of that suspension (see **Concentration- and residence-time-dependent rheology** below).

Spectral width of UV–vis attenuation spectra

To quantify the color homogeneity of the CNC films shown in Figure 1, their Full Width at Half Maximum values (FWHM, also referred to as spectral width) were estimated.

Table S2: Array of estimated spectral width values (in nanometers) from UV-Vis attenuation spectra. For spectra marked with "N/A", their peak wavelength was outside of the scanned wavelength range of 400 to 1100 nm, rendering it impossible to estimate their spectral width reliably.

	**0 J/mL**	**8 J/mL**	**30 J/mL**	**120 J/mL**	**480 J/mL**	**1440 J/mL**
**0 mmol/kg**	151	382	672	N/A	N/A	N/A
**150 mmol/kg**	206	161	100	181	240	N/A
**300 mmol/kg**	N/A	134	87	127	204	N/A

For [NaCl] = 0 mmol/kg, increasing sonication dose rapidly broadens the FWHM, indicating reflection over a wider range of wavelengths. For films at [NaCl] = 150 mmol/kg and [NaCl] = 300 mmol/kg, the FWHM appears to reach a minimum at u_s = 30 J/mL. Spectral width is correlated with disorder in the cholesteric structure. A broader range of reflected wavelengths is consistent with greater variation in cholesteric domain size, orientation, and pitch.

FCD-forming parameter window and reproducibility

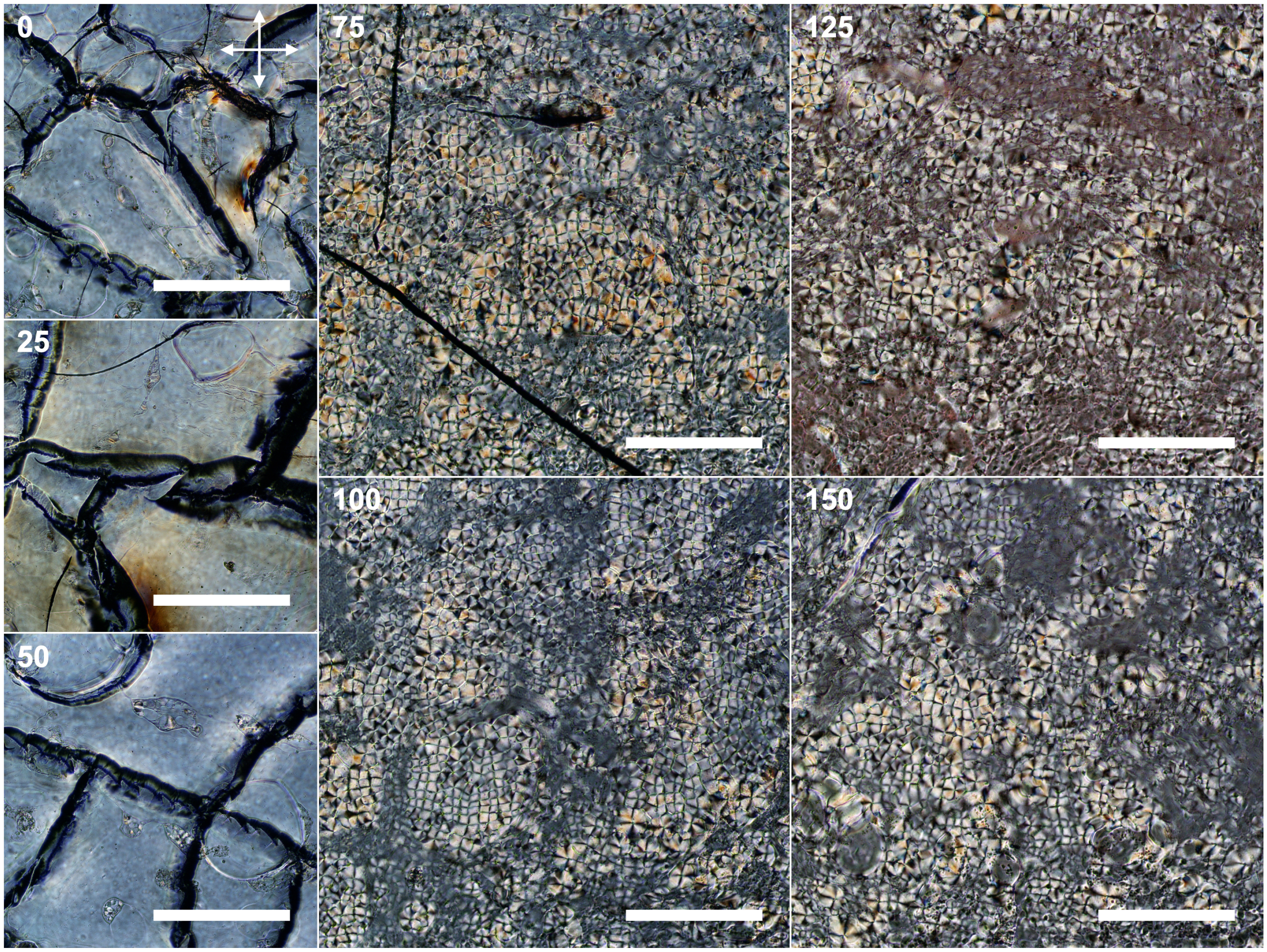

Figure S5: Polarized optical micrographs of CNC films prepared at a fixed sonication dose of $u_s = 120$ J/mL while varying [NaCl] from 0 to 150 mmol/kg CNC in increments of 25 mmol/kg. Films prepared with 0, 25, and 50 mmol/kg do not display characteristic FCD textures, whereas Maltese-cross textures associated with FCDs are observed from 75 to 150 mmol/kg. All images were acquired between crossed polarizers. Scale bars: 500 µm.

The initial parameter grid presented in Figure 2 used relatively large increments in salt concentration and sonication dose and identified [120 J/mL, 150 mmol/kg] as an FCD-rich condition. Closer inspection of the 150 mmol/kg series shows that FCDs are also present at lower sonication doses. FCDs are sparse at $u_s = 8$ J/mL, become more frequent at 30 J/mL, and are prominent throughout the film at 120 J/mL. Thus, within this series, sonication changes the abundance of FCDs over the range $u_s = 8$-120 J/mL.

To determine how this behavior extends across a broader processing window, we performed two additional parameter scans with smaller increments in [NaCl] and u_s. First, the sonication dose was fixed at $u_s = 120$ J/mL while [NaCl] was varied from 0 to 150 mmol/kg CNC in increments of 25 mmol/kg. Representative POM micrographs are shown in SI Figure S5. Films prepared with 0, 25, and 50 mmol/kg did not display the characteristic Maltese-cross textures associated with FCDs. In contrast, FCD textures were observed at 75, 100, 125, and 150 mmol/kg. These results show

that, at $u_s = 120$ J/mL, FCD formation occurs over a finite range of intermediate ionic content beginning between 50 and 75 mmol/kg.

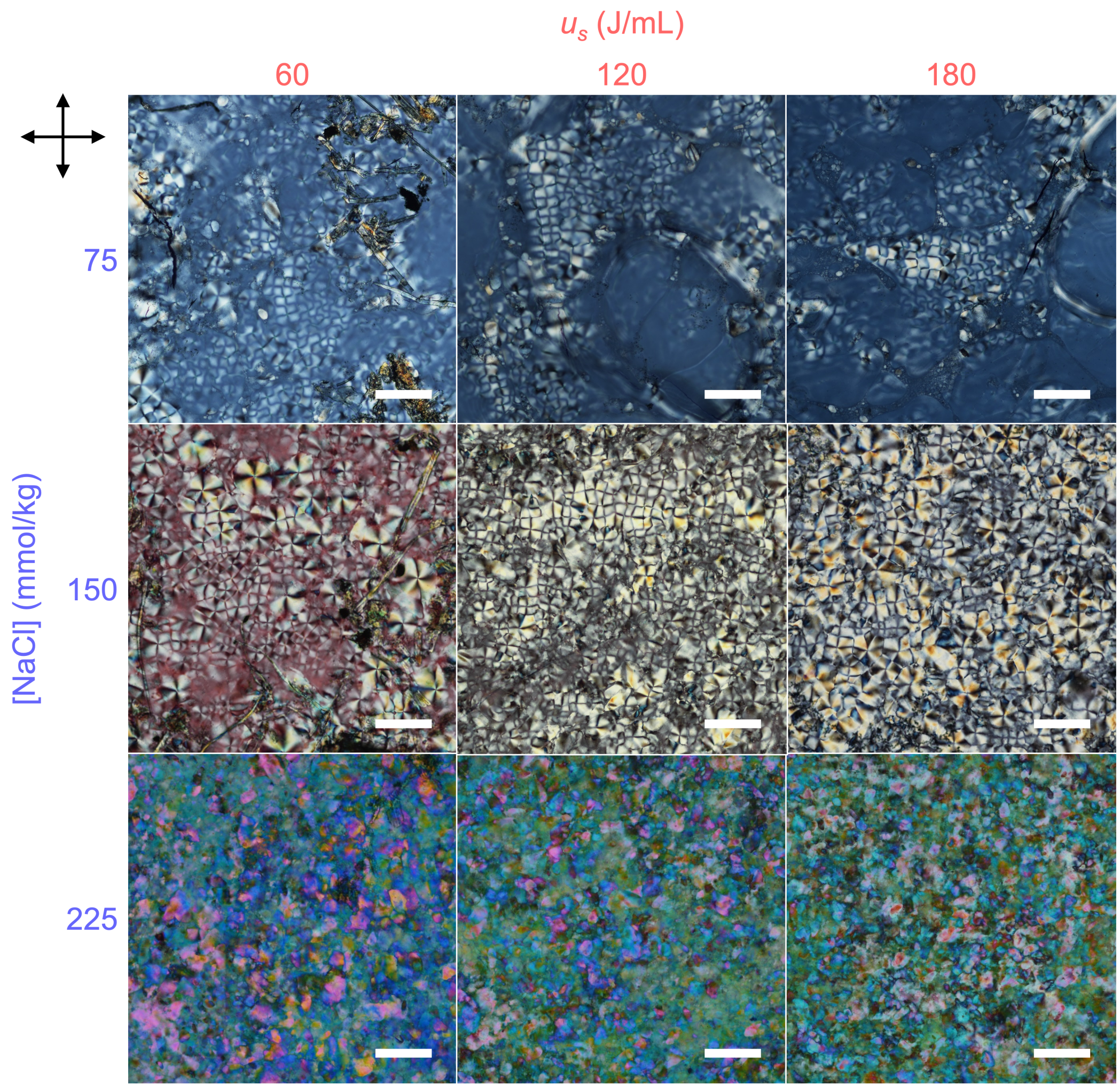

Figure S6: Polarized optical micrographs from a finer two-dimensional scan of the FCD-forming parameter space. Sonication dose increases from left to right, with $u_s = 60$, 120, and 180 J/mL, while [NaCl] increases from top to bottom, with concentrations of 75, 150, and 225 mmol/kg CNC. Characteristic FCDs are observed across multiple combinations at intermediate salt concentrations, whereas films prepared at 225 mmol/kg contain smaller and more disordered birefringent domains. All images were acquired between crossed polarizers. Scale bars: 50 µm.

A complementary two-dimensional parameter scan was performed using sonication doses of 60, 120, and 180 J/mL and [NaCl] values of 75, 150, and 225 mmol/kg CNC. As shown in SI Figure S6, characteristic FCDs were observed across several combinations of intermediate salt concentration and sonication dose. The films prepared at 75 and 150 mmol/kg contain regions with the characteristic fourfold Maltese-cross appearance of FCDs, although their size, density, and surrounding cholesteric texture vary with processing conditions. At 225 mmol/kg, the films instead contain smaller and more disordered birefringent domains, without similarly prominent FCD textures. Together with the increase in FCD abundance across the 150 mmol/kg series and the fixed-sonication salt scan, these observations show that FCD formation occurs within a finite

processing window governed jointly by salt concentration and sonication dose, while the prevalence and morphology of the FCDs vary continuously within this window.

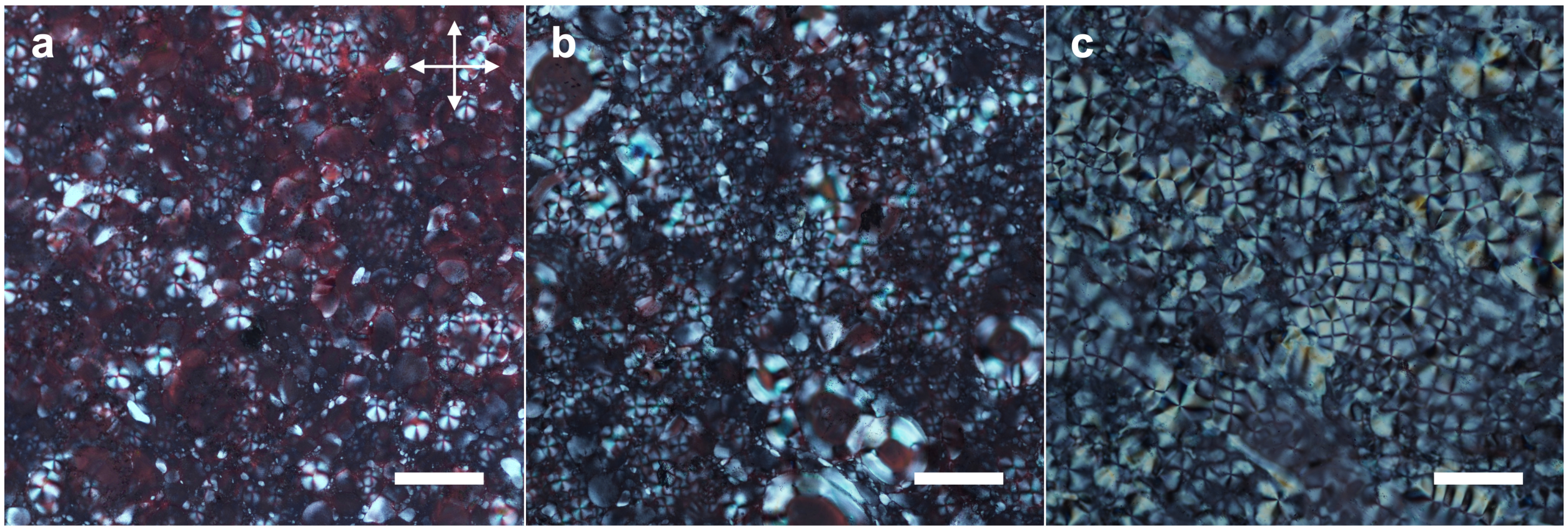

Figure S7: Reproducibility of FCD formation across independent CNC synthesis batches. Panels (a-c) show polarized optical micrographs of films prepared at [120 J/mL, 150 mmol/kg] using CNCs obtained from three separate sulfuric-acid-hydrolysis batches. Characteristic Maltese-cross textures are present in all three films, demonstrating that FCD formation under these processing conditions is not restricted to a single CNC batch. All images were acquired between crossed polarizers. Scale bars: 200 μm.

To test whether the FCD-forming behavior depended on a specific CNC synthesis, films were prepared at [120 J/mL, 150 mmol/kg] using CNCs obtained from three independent sulfuric-acid-hydrolysis batches. As shown in SI Figure S7, all three batches produced the characteristic Maltese-cross textures. Differences in the precise FCD size, density, color, and surrounding domain morphology are expected because of batch-to-batch variations in CNC dimensions, surface charge, aggregation state, and drying history. Nevertheless, the repeated formation of FCDs under the same salt and sonication conditions demonstrates that the phenomenon is reproducible across independently synthesized CNC materials.

[120 J/mL, 150 mmol/kg] film: slow evaporation

A film made from the [120 J/mL, 150 mmol/kg] suspension was slowly evaporated while under POM observation (see the time-lapse in SI Video 2). Its composition and evaporation method were identical to all other films, except it was loosely covered with a glass lid for the duration of the evaporation in order to slow it down. This extended the evaporation time from around 2 days (uncovered) to 11 days. In SI Figure S8, we show macro- and microscopic surface images of the produced film, its UV-Vis attenuation spectrum, and its exposed cross-section taken with SEM.

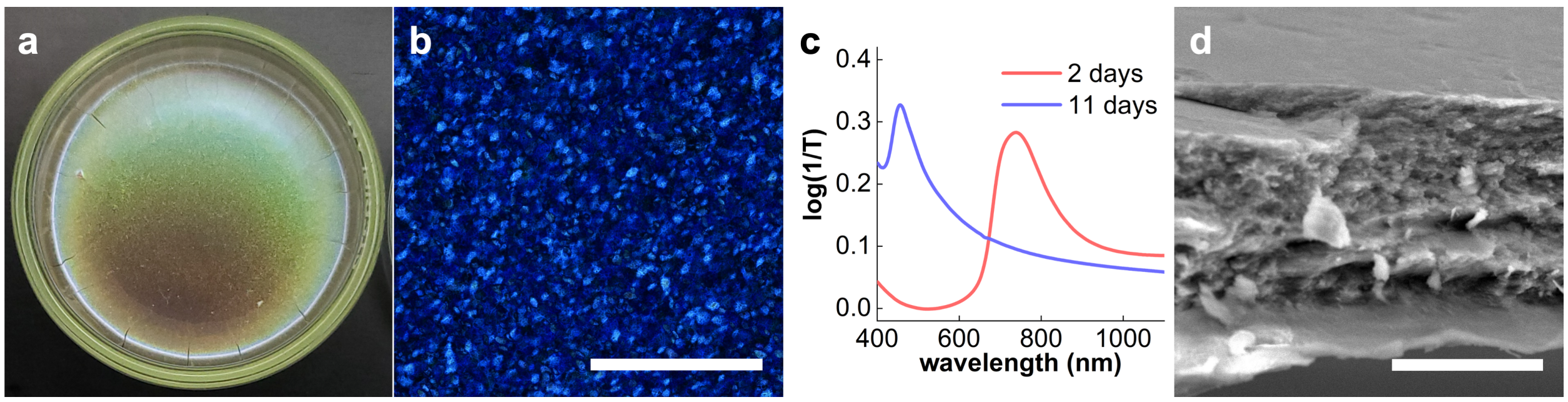

Figure S8: (a) Photograph of the entire dish-cast film. The petri dish diameter is 3.5 cm. (b) POM image of the film. (c) UV-Vis attenuation spectra comparing films evaporated from a [120 J/mL, 150 mmol/kg] suspension with differing evaporation times (2 and 11 days). (d) Cross-section image of the film, taken with SEM. Scale bars: b) 500 µm, d) 20 µm.

BARCODE analysis

Polarized optical microscopy time-lapse sequences were analyzed using the BARCODE framework[S8] to quantify structural and dynamic changes during evaporation. The BARCODE software and associated documentation are available through the BARCODE Github repository.[S9]

Binarization identifies connected optical regions, referred to here as islands. The number of islands reports the number of disconnected segmented regions, while the mean island area reports their average projected area relative to the field of view. During tactoid nucleation and growth, these quantities provide complementary measures of the number and characteristic size of the birefringent regions. We note that they are not direct physical tactoid counts once FCDs form because the alternating bright and dark sectors of a Maltese-cross texture can divide a single physical FCD into several segmented regions.

Optical flow quantifies frame-to-frame evolution of the POM texture. The mean speed is the spatially averaged magnitude of the optical-flow vectors and contains contributions from tactoid translation, growth, deformation, coalescence, and changes in internal texture. The speed standard deviation measures the spatial heterogeneity of this activity. Mean speed and speed standard deviation therefore distinguish broadly distributed structural motion from strongly localized rearrangement.

For event-aligned comparisons, the time axis was shifted so that either the first persistent appearance of cholesteric tactoids or the late speed-standard-deviation maximum associated with first clear FCD formation occurred at 0 min.

Absolute-time evolution at fixed ionic content

SI Figure S9 compares the absolute-time trajectories for the 0, 8, 30, and 120 J/mL samples at fixed [NaCl] = 150 mmol/kg. All four samples remain close to their isotropic baselines for a substantial fraction of the drying sequence before the rapid appearance of cholesteric structure. The subsequent pathways, however, differ markedly.

The maximum island population (SI Figure S9a) increases overall with sonication dose, with the largest tactoid-associated population occurring for the FCD-rich 120 J/mL condition. The unsonicated sample nevertheless develops several hundred segmented regions, demonstrating that a substantial tactoid population is not by itself sufficient for FCD formation.

The mean island area (SI Figure S9b) provides an important complementary comparison. The unsonicated sample develops the largest mean island areas and continues to coalesce over a long interval after its initial island population has decreased. By contrast, the sonicated samples generate more numerous, generally smaller segmented regions over shorter intervals. Thus, island number and mean island area together show that sonication primarily regulates how rapidly the emerging cholesteric phase subdivides and consolidates into a coherent precursor, while the largest final domains are observed under conditions that do not form abundant FCDs.

Mean speed and speed standard deviation (SI Figure S9c and SI Figure S9d, respectively) further distinguish these pathways. The principal motion peaks occur during the tactoid growth and coalescence intervals. Later features in the speed standard deviation occur after much of the broad tactoid motion has subsided and, for the FCD-containing samples, coincide with the appearance of focal-conic textures.

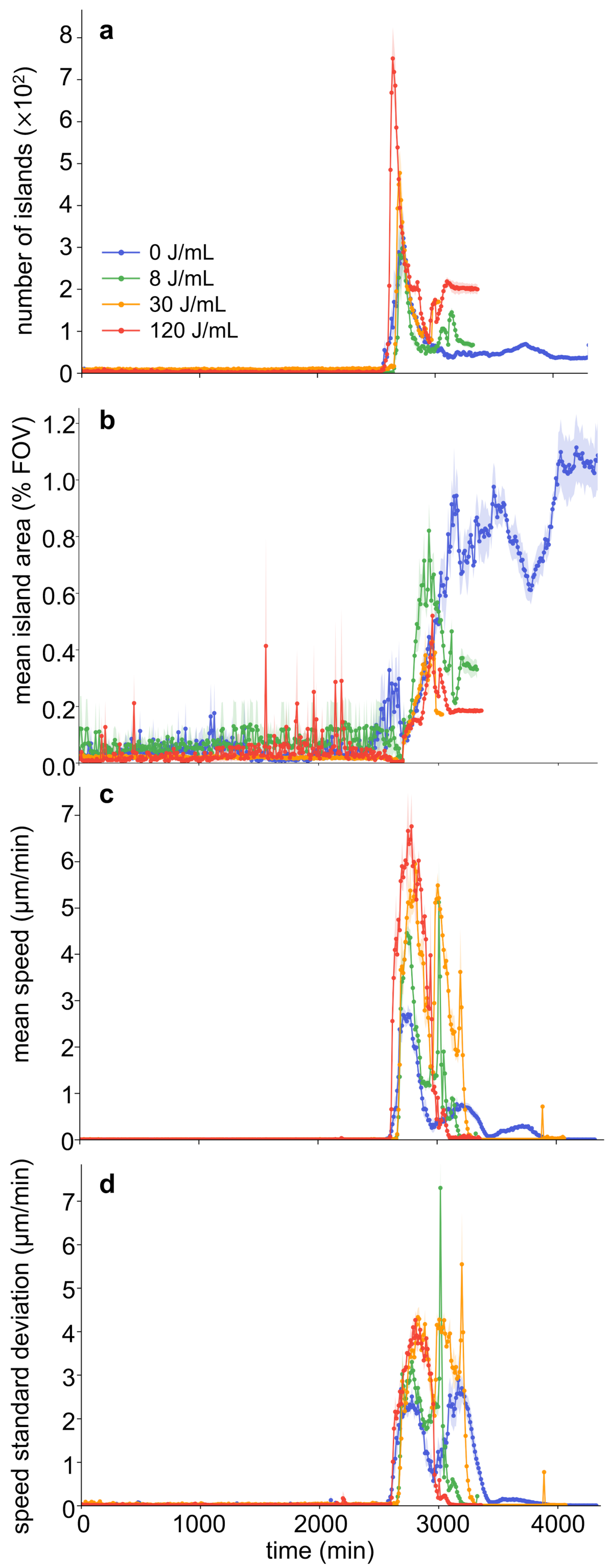

Figure S9: Absolute-time BARCODE analysis of CNC suspensions containing 150 mmol NaCl/kg CNC and prepared at sonication doses of 0, 8, 30, and 120 J/mL. The panels show a) number of islands, b) mean island area, c) mean speed, and d) speed standard deviation. Solid lines show values averaged over four image quadrants, and shaded regions indicate the corresponding inter-quadrant variability.

Event-aligned dynamics at fixed ionic content

To compare the kinetics following first tactoid appearance directly, the 150 mmol/kg trajectories were shifted such that $t_{\mathrm{nuc}} = 0$ corresponds to the onset of persistent cholesteric tactoids (SI Figure S10a-d). This representation emphasizes that increasing sonication compresses the early assembly pathway.

The maximum island population increases overall from the unsonicated condition toward 120 J/mL, while the time required to reach this maximum decreases strongly (SI Figure S10a,e). The unsonicated sample develops a tactoid population comparable to the low-sonication condition but requires substantially longer to reach this state. Increasing FCD abundance is therefore more closely associated with rapid generation and coalescence of the tactoid population than with island number alone.

The mean island area evolves on a longer timescale than island number (SI Figure S10b,f). Most notably, the non-FCD-forming unsonicated sample continues to coalesce and ultimately develops the largest mean island area. This prolonged growth may reflect differences in the evolving rheological and microstructural state of the suspension during concentration. The comparatively low apparent viscosity measured for the unsonicated sample (see **Concentration- and residence-time-dependent rheology** below) could permit tactoid growth and coalescence over a longer interval, while its larger particle and bundle population may also influence the characteristic domain size. By comparison, the FCD-rich 120 J/mL pathway is characterized by rapid generation of a large tactoid population followed by rapid coalescence into an extended cholesteric precursor before the later FCD-forming event.

The mean-speed trajectories identify the interval of strongest tactoid-scale structural activity (SI Figure S10c,g). The speed-standard-deviation trajectories show that the spatial heterogeneity of this activity evolves differently from its mean magnitude (SI Figure S10d,h). In the FCD-containing samples, the later increase in speed standard deviation occurs after the primary tactoid-motion interval and corresponds to the onset of FCDs.

The structural state immediately preceding this event is further summarized in SI Figure S10i-k. The island population decreases substantially between its nucleation-associated maximum and the later FCD stage, while the characteristic island area increases, consistent with tactoid growth and coalescence before FCD formation. At FCD onset, island number increases again while mean island area decreases, reflecting in part the subdivision of the newly developed Maltese-cross texture into multiple segmented regions. The time required to reach the intervening minimum island population decreases with increasing sonication dose, providing an additional measure of increasingly rapid tactoid coalescence.

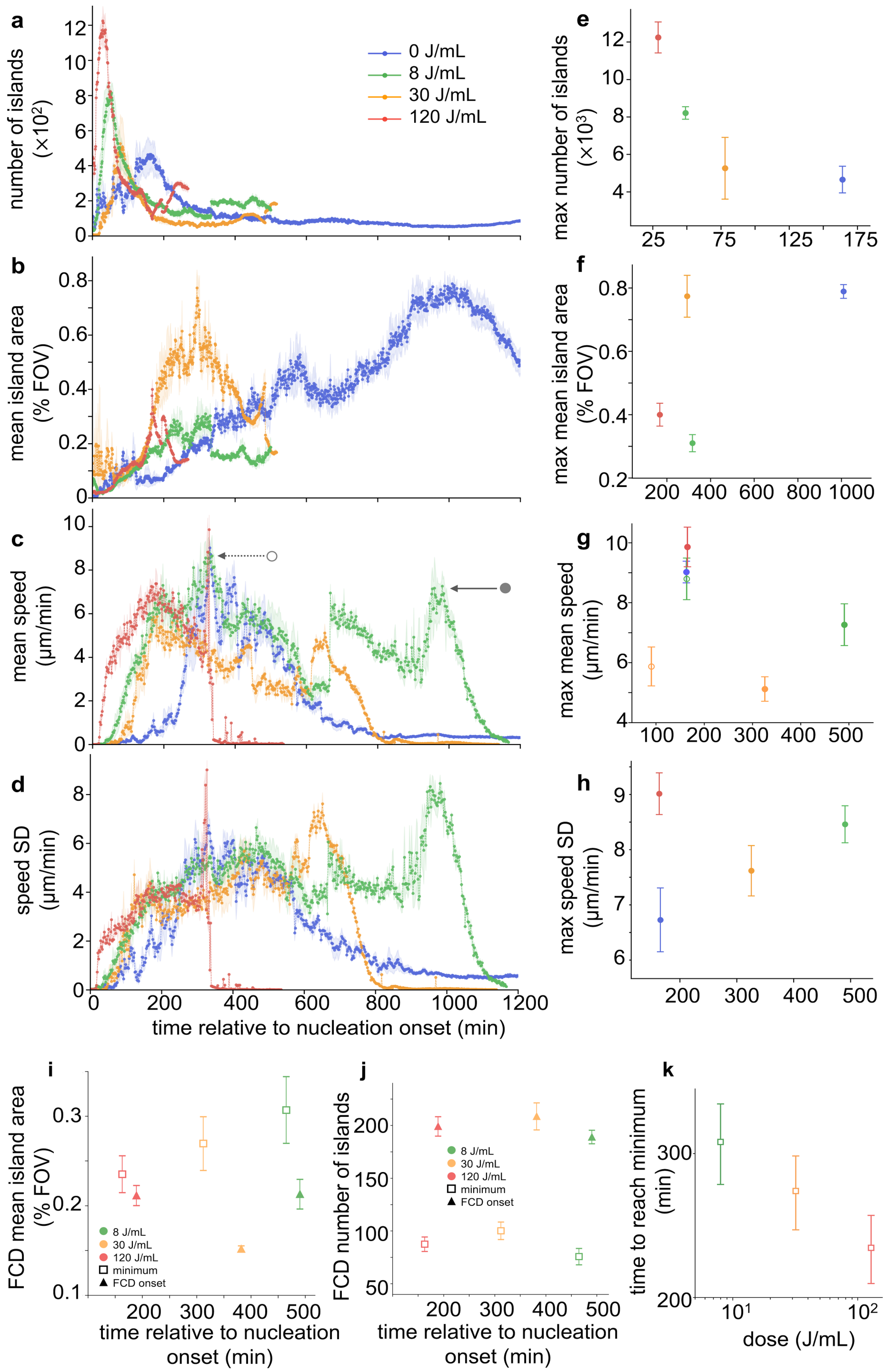

Figure S10: Event-aligned BARCODE analysis of CNC suspensions containing 150 mmol NaCl/kg CNC. Time is plotted relative to first persistent tactoid appearance. (a-d) Time-dependent number of islands, mean island area, mean speed, and speed standard deviation for the 0, 8, 30, and 120 J/mL conditions. (e-h) Maximum values of the corresponding descriptors plotted against the time required to reach those maxima. (i,j) Mean island area and number of islands at the minimum-island stage and at FCD onset for the FCD-containing 8, 30, and 120 J/mL samples. Open squares denote the minimum-island stage and filled triangles denote FCD onset. (k) Time required to reach the minimum island population as a function of sonication dose.

Comparison with extreme processing conditions

SI Figure S11 compares [1440 J/mL, 0 mmol/kg], [0 J/mL, 450 mmol/kg], and [8 J/mL, 150 mmol/kg] to test whether individual structural or dynamic signatures are sufficient for FCD formation.

The salt-free/high-sonication and unsonicated/high-salt samples enter the birefringent regime substantially earlier than the [8 J/mL, 150 mmol/kg] sample, shown through the number of islands plotted against time in SI Figure S11a. Both generate large island populations and undergo substantial structural motion, despite not forming prominent FCD textures. The high-salt [0 J/mL, 450 mmol/kg] sample also develops very large mean island areas (SI Figure S11b), further demonstrating that the production of large cholesteric regions is not sufficient to generate the focal-conic morphology.

The amplitudes of the mean-speed and speed-standard-deviation responses overlap substantially among these conditions (see SI Figure S11c and SI Figure S11d). Consequently, neither strong motion nor spatially heterogeneous motion alone identifies the FCD-forming pathway. The relevant distinction instead lies in the sequence and timing of tactoid nucleation, growth, coalescence, and the later FCD-associated event.

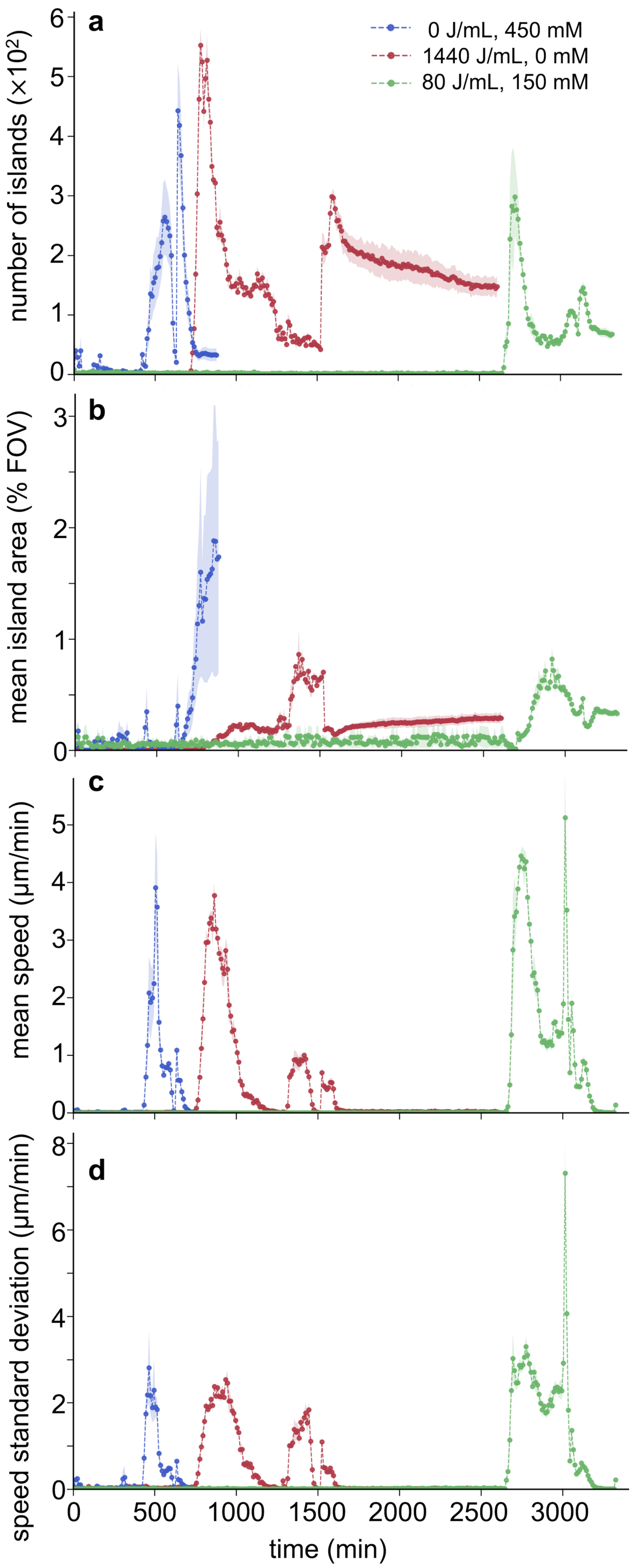

Figure S11: BARCODE comparison of [1440 J/mL, 0 mmol/kg], [0 J/mL, 450 mmol/kg], and [8 J/mL, 150 mmol/kg]. The panels show a) number of islands, b) mean island area, c) mean speed, and d) speed standard deviation as a function of physical drying time. Solid lines show values averaged over four image quadrants, and shaded regions indicate the corresponding inter-quadrant variability.

Effect of evaporation rate at fixed composition

The standard and slow evaporation conditions for the [120 J/mL, 150 mmol/kg] formulation, introduced in SI Figure S8, were further compared using BARCODE analysis in SI Figure S12. Composition, substrate, and nominal anchoring conditions were held fixed, while partial covering increased the drying time from approximately 2 to 11 days.

Under standard evaporation, cholesteric tactoids appear, rapidly grow and coalesce, and subsequently develop the characteristic focal-conic texture. Under slow evaporation, the suspension remains isotropic for a much longer period and subsequently develops a large population of comparatively small tactoids that coalesce only gradually. The final film remains dominated by incompletely coalesced domains and domain walls.

The BARCODE descriptors quantify this difference. Standard evaporation produces a rapid increase and subsequent decrease in island number together with a pronounced increase in mean island area during tactoid coalescence. Slow evaporation produces a substantially larger peak island population followed by a prolonged decrease, while the mean island area remains comparatively small and increases only gradually. The slow-drying pathway therefore favors the persistence of numerous small domains instead of rapid coalescence into the larger connected cholesteric precursor observed under standard conditions.

The dynamic descriptors show a corresponding distinction. Standard evaporation produces a high-amplitude, temporally concentrated mean-speed response together with the sharp increase in speed standard deviation associated with FCD formation. Under slow evaporation, mean speed is lower and distributed over a much longer interval, while speed heterogeneity remains comparatively small and changes gradually. The localized dynamical response associated with FCD formation under standard evaporation is therefore strongly suppressed when the drying rate is reduced.

These observations support a kinetic interpretation of the evaporation-rate dependence. Slower evaporation allows structural evolution to proceed through gradual growth and rearrangement of many small tactoids, limiting their rapid coalescence into a spatially coherent cholesteric region. Under standard evaporation, rapid tactoid growth and coalescence instead generate an extended layered precursor while continued solvent loss changes the preferred cholesteric spacing and film thickness. The suppression of both this rapid coalescence pathway and the late FCD-associated dynamical event under slow evaporation is consistent with reduced access to the collective layer deformation associated with a Helfrich-Hurault-like instability.

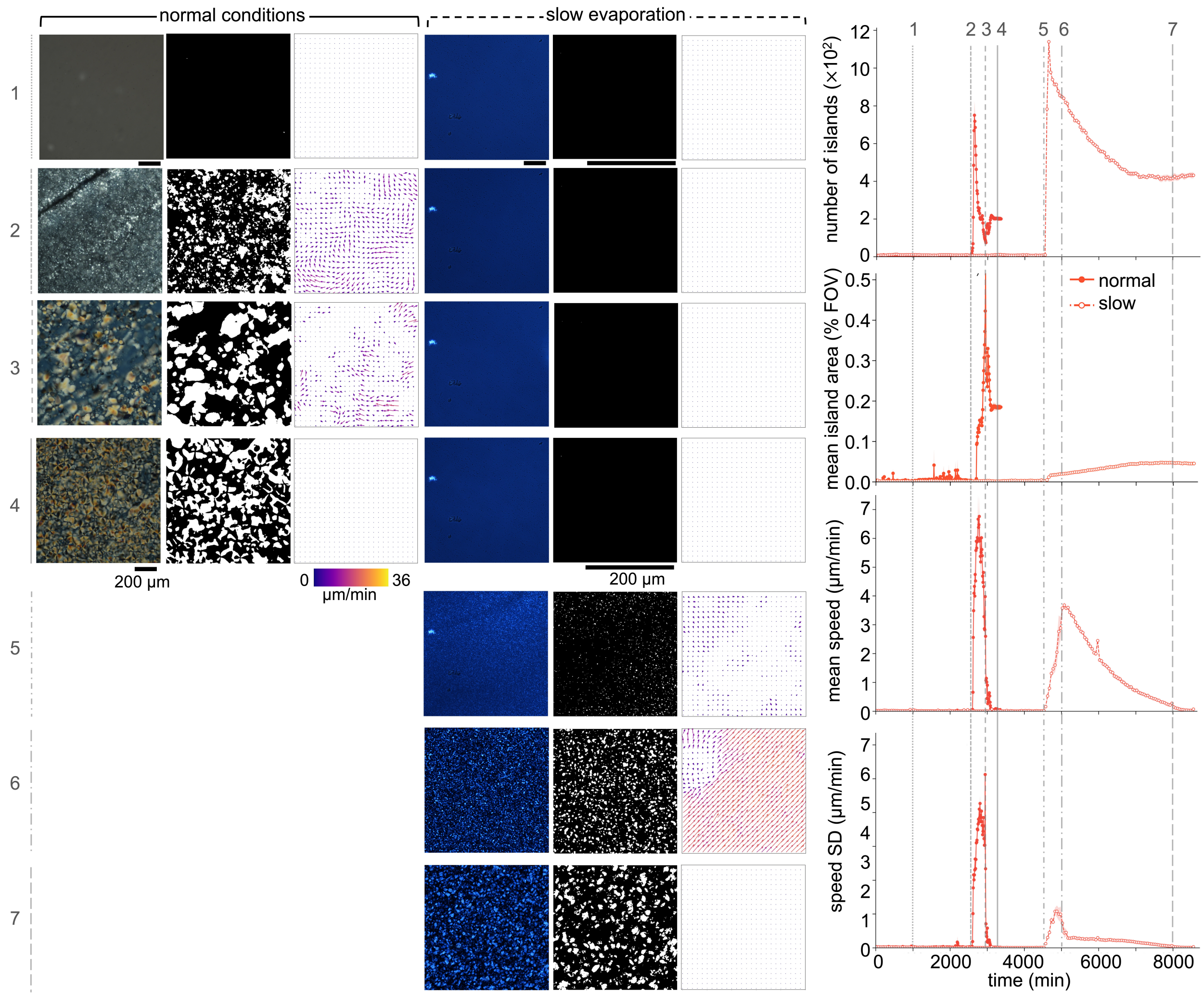

Figure S12: Comparison of standard and slow evaporation for the [120 J/mL, 150 mmol/kg] formulation. Representative POM images at the numbered stages are shown on the left, with the corresponding event times marked on the BARCODE trajectories on the right. The quantitative panels show mean island area, number of islands, speed standard deviation, and mean speed. Solid symbols and lines denote standard evaporation and open symbols and lines denote slow evaporation. Scale bars: 200 μm.

Concentration- and residence-time-dependent rheology

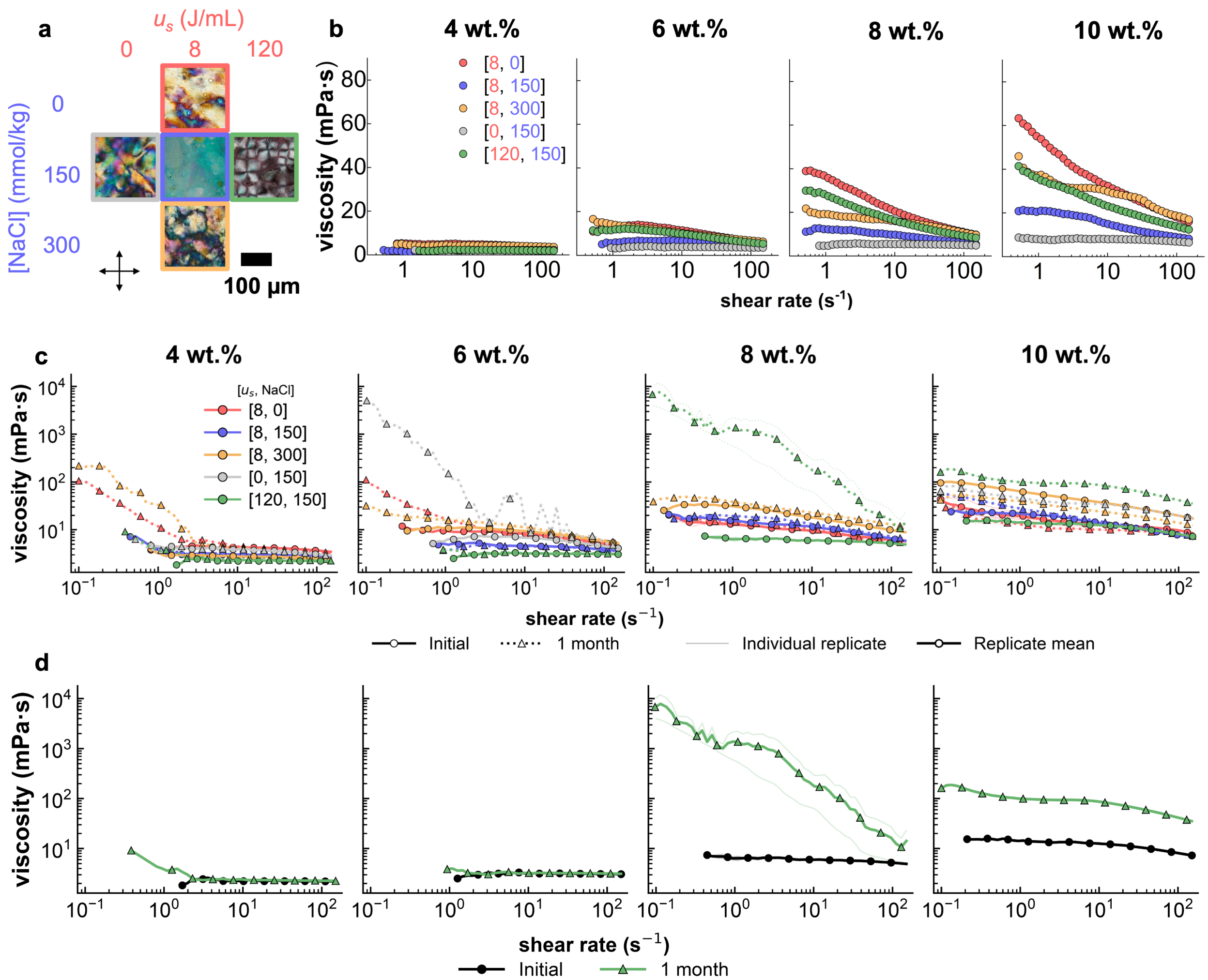

Figure S13: Concentration- and residence-time-dependent rheology of representative CNC suspensions. (a) POM images of films prepared from five salt-sonication conditions, reproduced from Figure 2. The colored outlines correspond to the sample colors used in the rheological plots. (b) Flow curves measured at 4, 6, 8, and 10 wt.% CNC, from left to right, approximately three days after preparation at the target CNC concentrations. (c) Separate aging measurements comparing initial and one-month flow curves for all five salt-sonication conditions at 4, 6, 8, and 10 wt.% CNC. Initial measurements are shown using solid lines and circular markers, while one-month measurements are shown using dotted lines and triangular markers. Thin curves show individual replicate measurements, and the more prominent curves show the replicate means. (d) Initial and one-month flow curves from the aging measurements for the [120 J/mL, 150 mmol/kg] suspension, shown separately at each CNC concentration. Initial measurements are shown in black and one-month measurements in green. Age denotes residence time at the specified CNC concentration.

Rheological measurements were performed to determine how the apparent viscosity of representative CNC suspensions depends on salt concentration, sonication dose, CNC concentration, shear rate, and residence time at fixed concentration. Five processing conditions were selected to represent the range of film morphologies observed in the main parameter grid: [8 J/mL, 0 mmol/kg], [8 J/mL, 150 mmol/kg], [8 J/mL, 300 mmol/kg], [0 J/mL, 150 mmol/kg], and [120 J/mL, 150 mmol/kg]. The corresponding POM images are reproduced in SI Figure S13a to allow comparison between the suspension flow response and the final film morphology.

The concentration-series flow curves in SI Figure S13b were measured approximately three days after preparation at the target CNC concentrations. Apparent viscosity generally increases with CNC concentration and decreases with increasing shear rate. The latter behavior is consistent with shear-induced alignment and disruption of concentration-dependent structures in suspensions of rod-like CNCs.[S10,S11] The magnitude and shape of the flow curves vary nonmonotonically with salt concentration and sonication dose, indicating distinct concentration- and processing-dependent suspension responses.

For suspensions prepared at $u_s = 8$ J/mL, the sample containing 150 mmol NaCl/kg CNC has a lower apparent viscosity than the corresponding 0 and 300 mmol/kg samples at 8 and 10 wt.% CNC. This nonmonotonic salt dependence is consistent with competition between electrostatic screening, reduction of the effective interaction volume, and association at higher ionic content.[S12] At 150 mmol/kg, the apparent viscosity also varies with sonication dose. Sonication can reduce viscosity through fragmentation of aggregates,[S10,S13] while the resulting changes in particle number density, aggregate and tactoid populations, orientational organization, and network connectivity can further alter the suspension response. The observed flow curves therefore reflect multiple changes in suspension microstructure.

Empirical power-law description of the concentration-series flow curves

To compare the shear-rate dependence of the five suspensions using a common set of empirical parameters, the 8 wt.% concentration-series flow curves in SI Figure S13b were fitted to the Ostwald-de Waele power-law model:[S14]

$$\mu = K\dot{\gamma}^{n-1}, \tag{4}$$

where μ is the apparent viscosity, $\dot{\gamma}$ is the applied shear rate, K is the flow consistency index, and n is the flow behavior index. The 8 wt.% concentration was selected because differences among the five processing conditions are clearly resolved while all samples remain measurable over a substantial common shear-rate interval. Within this empirical description, K sets the approximate viscosity scale and $n < 1$ describes a viscosity that decreases with increasing shear rate. A smaller value of n corresponds to a steeper shear-rate dependence.

The power-law model provides a compact empirical description of monotonic shear thinning over the measured interval. Concentrated, partially liquid-crystalline CNC suspensions may additionally contain multiple structural populations or undergo changes in flow regime that are not represented by a single power law. Deviations from the model can therefore reflect a more complex shear-dependent suspension response.

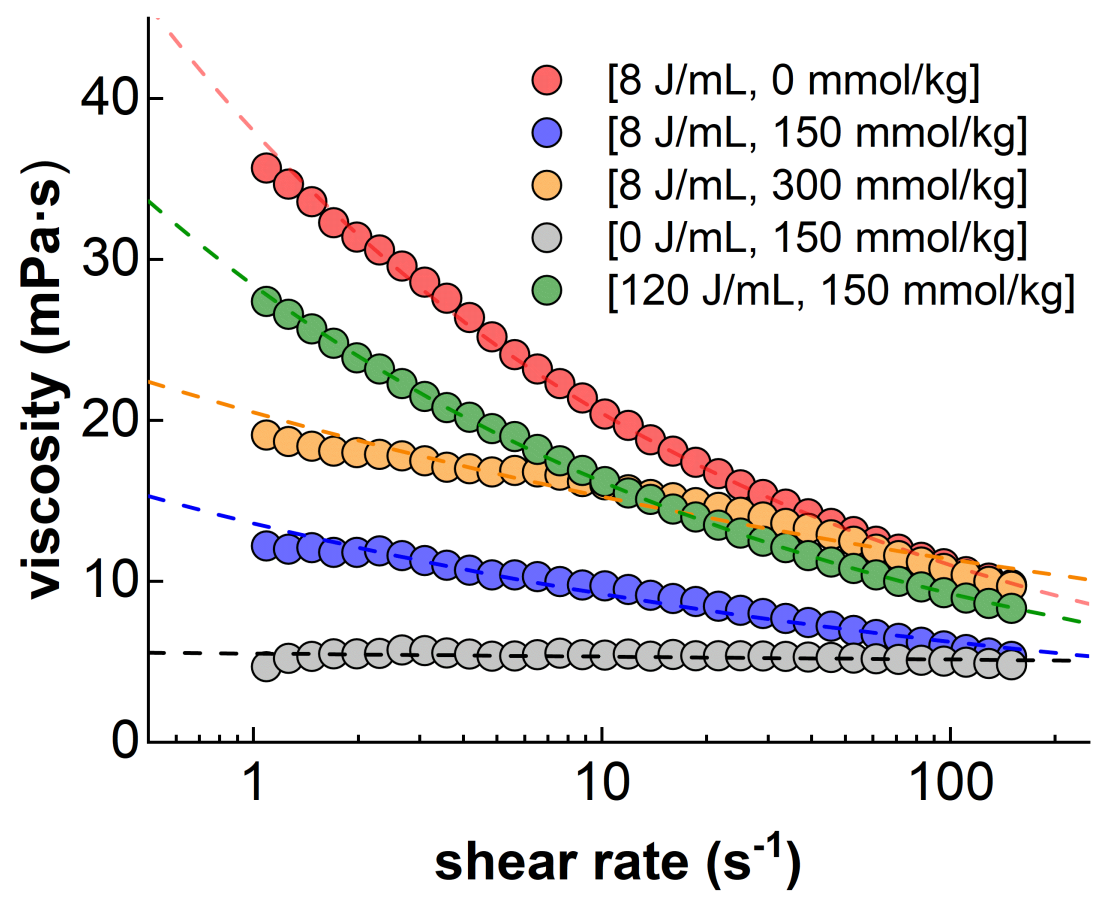

Figure S14: Apparent viscosity of the 8 wt.% CNC suspensions from the concentration-series measurements in SI Figure S13b as a function of shear rate. Experimental data are shown as circles and empirical power-law fits obtained using Equation 4 are shown as dashed lines. The corresponding flow consistency indices, flow behavior indices, and coefficients of determination are summarized in SI Table S3.

Table S3: Empirical power-law parameters obtained from the 8 wt.% concentration-series flow curves shown in SI Figure S14.

**Sample**	K **(Pa·s^n)**	n	R^2
[8 J/mL, 0 mmol/kg]	0.038	0.731	0.9946
[8 J/mL, 150 mmol/kg]	0.017	0.831	0.9640
[8 J/mL, 300 mmol/kg]	0.021	0.872	0.9372
[0 J/mL, 150 mmol/kg]	0.005	0.985	0.2457
[120 J/mL, 150 mmol/kg]	0.028	0.756	0.9995

The fitted values of n are below unity for all five samples, consistent with the observed decrease in apparent viscosity with increasing shear rate. The [8 J/mL, 0 mmol/kg] and [120 J/mL, 150 mmol/kg] suspensions show the strongest fitted shear-rate dependence, with $n = 0.731$ and 0.756, respectively. The [0 J/mL, 150 mmol/kg] suspension gives $n = 0.985$, nominally close to a shear-rate-independent response, although the low coefficient of determination ($R^2 = 0.2457$) shows that a single power law does not adequately describe its flow curve.

The deviation of the unsonicated suspension from the power-law model is consistent with a heterogeneous population of CNC aggregates and other mesoscale structures. Different structural populations may contribute to the measured response over different shear-rate intervals, giving a more complex curve than can be represented by single values of K and n.

Effect of aggregate removal on film morphology

To examine the role of large aggregates independently of tip sonication, films were prepared from two portions of the same [0 J/mL, 150 mmol/kg] suspension. Before drop casting, one portion was passed through a cellulose-acetate syringe filter with a nominal pore size of 0.8 µm to reduce the population of large aggregates. The second portion was used without filtration. The resulting film textures are compared in SI Figure S15.

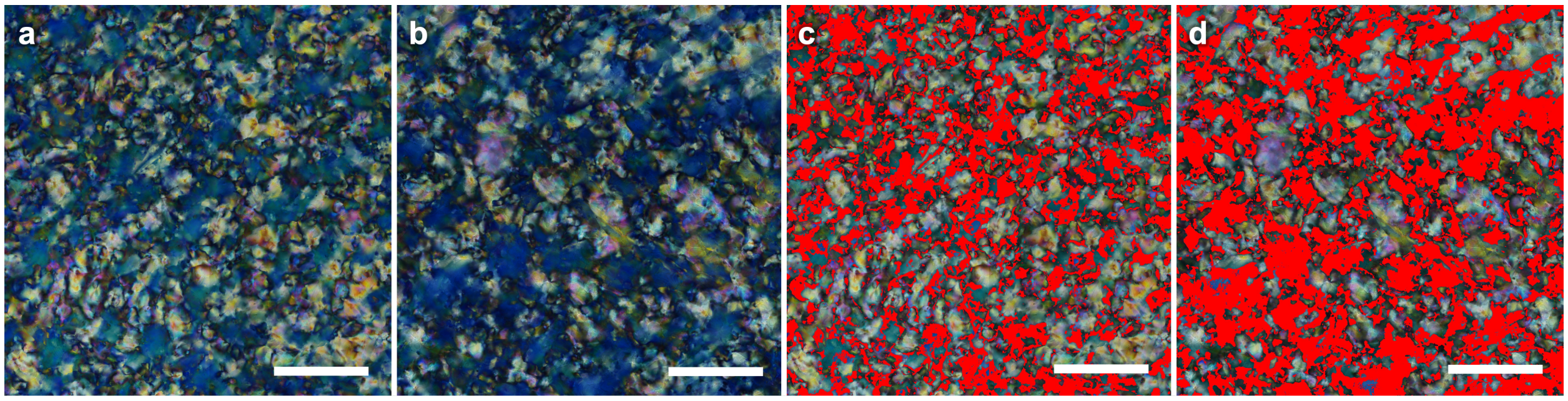

Figure S15: Stitched POM micrographs of films prepared from (a) unfiltered and (b) filtered [0 J/mL, 150 mmol/kg] CNC suspensions. Panels (c) and (d) show the same fields of view as (a) and (b), respectively, with pixels falling within a selected blue-color range displayed in red. The same ImageJ threshold was applied to both images. Scale bars: 500 µm.

The filtered film contains larger and more spatially continuous blue regions than the unfiltered film. To compare the images using the same color criterion, pixels were selected in ImageJ using hue values from 140 to 200, saturation values from 140 to 255, and brightness values from 0 to 140. The selected pixels account for 31% of the analyzed area in the unfiltered film and 42% in the filtered film. This color-threshold analysis quantifies the increase in area occupied by the selected blue tones following filtration.

Together with the AFM and DLS measurements showing that sonication decreases CNC particle and bundle size (SI Figures S3 and S4), the filtration experiment indicates that large aggregates contribute to spatial heterogeneity in the final film. Aggregate population and apparent viscosity therefore describe different aspects of the suspension state. The [0 J/mL, 150 mmol/kg] suspension combines comparatively low apparent viscosity with a heterogeneous particle and bundle population and produces incomplete or nonuniform tactoid coalescence.

Residence-time-dependent evolution

The aging measurements in SI Figure S13c compare the initial flow response with that measured after one month of residence at each fixed CNC concentration. Rheological aging depends strongly on both processing condition and CNC concentration. Different salt-sonication conditions exhibit pronounced changes at different concentrations, and the individual replicate curves vary in the magnitude of their response. The replicate means nevertheless reveal systematic concentration-dependent evolution for several conditions.

The [120 J/mL, 150 mmol/kg] suspension, which produces the FCD-rich films examined in the main text, is shown separately in SI Figure S13d. At 4 and 6 wt.% CNC, the initial and one-month mean curves remain comparatively similar over most of the measured shear-rate range. At 8 wt.%, the one-month sample exhibits an orders-of-magnitude increase in low-shear apparent viscosity and a much stronger decrease in viscosity with increasing shear rate. A substantial aging response is also present at 10 wt.%, where the one-month curve remains above the initial curve throughout the measured range.

The power-law framework provides a qualitative description of the changes in curve shape. At 8 wt.%, the strong elevation at low shear rate followed by convergence toward the initial curve at high shear rate corresponds qualitatively to a higher effective consistency and stronger shear-rate dependence after one month. This response is consistent with a structure that strongly resists weak deformation and is progressively disrupted or aligned as the shear rate increases. At 10 wt.%, the

upward displacement of the one-month curve across the full shear-rate interval indicates an increase in overall consistency, while the change in slope is less pronounced than at 8 wt.%.

Several aged suspensions also exhibit curvature, replicate variability, and changes in slope across the measured interval, consistent with contributions from multiple structural populations or shear-induced rearrangements. These curve shapes are therefore discussed qualitatively rather than represented by a single set of power-law parameters.

For the [120 J/mL, 150 mmol/kg] suspension, the particularly strong aging response near 8 wt.% CNC indicates that persistent mesoscale organization becomes readily accessible at this concentration. At lower concentrations, CNC-rich regions, tactoids, or aggregates may remain more weakly associated, producing comparatively small changes during residence at fixed concentration. At higher concentrations, more frequent and longer-lived contacts can promote orientationally correlated structures, tactoid association, or formation of a shear-sensitive network. Such structures are consistent with the high low-shear viscosity and pronounced shear thinning observed after one month.

The aging measurements characterize structural evolution during extended residence at fixed CNC concentration, whereas film formation involves continuous concentration during evaporation. The drying suspension therefore passes through each concentration interval on a much shorter timescale. These measurements establish that the rheological state depends strongly on both CNC concentration and residence time and provide complementary information about the evolving capacity of the suspension for structural rearrangement.

Density contrast and buoyancy-associated flow

The linear tactoid arrangements and apparent recirculating motion observed during evaporation are consistent with buoyancy-associated transport during isotropic-cholesteric phase separation. To determine the sign and approximate magnitude of the phase-density contrast, 4 mL of a [30 J/mL, 150 mmol/kg] suspension at 12 wt.% CNC was allowed to equilibrate for two weeks. The resulting sample contained approximately equal volumes of isotropic and cholesteric phases. A 100 µL aliquot of the upper isotropic phase had a mass of 103.2 mg, corresponding to $\rho_{\text{iso}} = 1.032$ g/mL, while an equal volume of the lower cholesteric phase had a mass of 106.5 mg, corresponding to $\rho_{\text{chol}} = 1.065$ g/mL. The measured density difference was therefore $\Delta\rho = 0.033$ g/mL, or 33 kg/m^3, with the cholesteric phase being denser under these equilibrium conditions.

The measured density contrast between the isotropic and cholesteric phases establishes a buoyancy force that can contribute to the recirculating motion observed during phase separation. Quantitative prediction of convective onset is complicated by the continuously evolving state of the drying suspension. The liquid-layer depth decreases with time, CNC concentration increases, and the suspension becomes increasingly anisotropic and shear thinning as the cholesteric phase develops. The relevant viscosity and collective transport coefficients therefore also evolve throughout drying. We describe the observed recirculation as convection-like or buoyancy-associated and assess its contribution to assembly using the time-lapse observations and the controlled change in evaporation rate.

Spectral bandwidth and angular dispersion

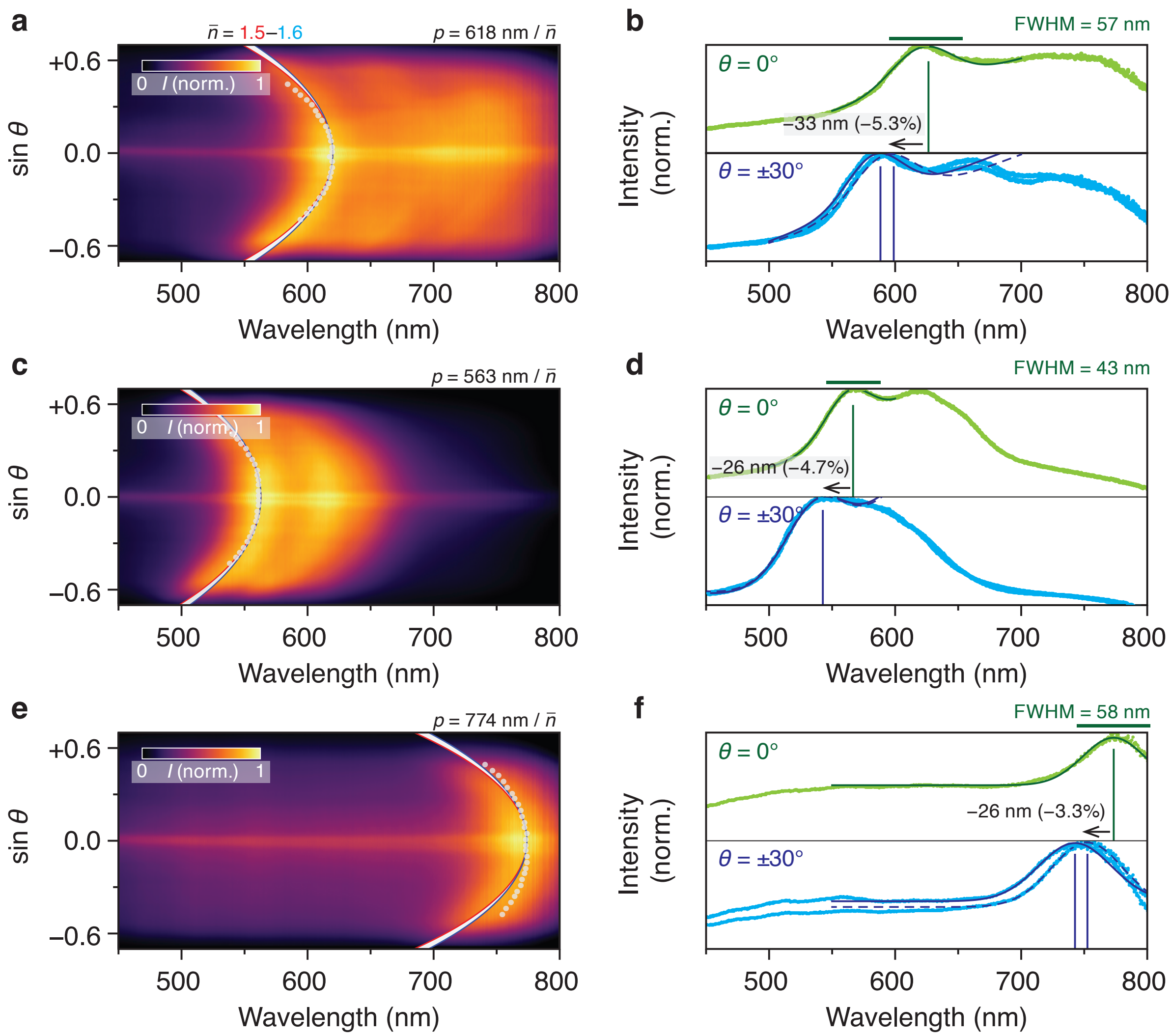

Figure S16: Quantitative comparison of the local spectral bandwidth and angular dispersion of three representative CNC films. Panels (a), (c), and (e) show angle-resolved reflection maps for the [8 J/mL, 0 mmol/kg], [8 J/mL, 150 mmol/kg], and FCD-rich [120 J/mL, 150 mmol/kg] films, respectively. The overlaid curves delimit the locally planar cholesteric responses calculated using $\overline{n} = 1.5\text{-}1.6$. For each calculated response, the pitch was set by $p = \lambda(0^\circ)/\overline{n}$, giving $p = 618\ \mathrm{nm}/\overline{n}$, $563\ \mathrm{nm}/\overline{n}$, and $774\ \mathrm{nm}/\overline{n}$ for panels (a), (c), and (e), respectively. Panels (b), (d), and (f) show wavelength cross-sections at $\theta = 0^\circ$ and $\pm 30^\circ$. The two spectra measured at -30° and $+30^\circ$ are shown together in blue. Gaussian fits to the spectra at $\theta = 0^\circ$ give FWHM values of 57, 43, and 58 nm for panels (b), (d), and (f), respectively. The signed wavelength shifts indicated in the panels are the averages of the shifts measured from 0° to -30° and from 0° to $+30^\circ$. The resulting mean shifts are -33, -26, and -26 nm, corresponding to fractional shifts of -5.3, -4.7, and -3.3%, respectively. Negative values indicate blue shifts.

To compare the local spectral bandwidth and angular response of films with different central reflection wavelengths, three representative samples were analyzed in greater detail: [8 J/mL, 0 mmol/kg], [8 J/mL, 150 mmol/kg], and the FCD-rich [120 J/mL, 150 mmol/kg] film. The angle-

resolved reflection maps are shown in SI Figure S16a,c,e, and the corresponding wavelength cross-sections at the microscope-defined external angles $\theta = 0^\circ$ and $\pm 30^\circ$ are shown in SI Figure S16b,d,f.

The peak wavelength measured at the microscope-defined central angle is denoted $\lambda(0^\circ)$. The signed mean wavelength shift at $\pm 30^\circ$ was calculated as

$$\langle \Delta\lambda \rangle_{\pm 30^\circ} = \frac{1}{2} \left\{ [\lambda(+30^\circ) - \lambda(0^\circ)] + [\lambda(-30^\circ) - \lambda(0^\circ)] \right\}. \quad (5)$$

Negative values of $\langle \Delta\lambda \rangle_{\pm 30^\circ}$ indicate a blue shift. The corresponding mean fractional wavelength shift was calculated as

$$\langle \delta \rangle_{\pm 30^\circ} = \frac{\langle \Delta\lambda \rangle_{\pm 30^\circ}}{\lambda(0^\circ)}. \quad (6)$$

Averaging the spectra measured at -30° and $+30^\circ$ reduces the influence of minor asymmetry caused by small deviations of the film surface from perfect alignment with the microscope stage. The angle θ refers to the external angle defined relative to the microscope objective.

Table S4: Spectral bandwidth and angular-shift metrics obtained from SI Figure S16. The signed mean wavelength shift is defined by Equation 5, and the corresponding mean fractional shift is defined by Equation 6. Negative values indicate blue shifts.

Condition	$\lambda(0^\circ)$ (nm)	$\langle \Delta\lambda \rangle_{\pm 30^\circ}$ (nm)	$\langle \delta \rangle_{\pm 30^\circ}$ (%)	FWHM (nm)
[8 J/mL, 0 mmol/kg]	618	−33	−5.3	57
[8 J/mL, 150 mmol/kg]	563	−26	−4.7	43
[120 J/mL, 150 mmol/kg]	774	−26	−3.3	58

Comparison of the absolute wavelength displacement alone does not fully describe the relative angular dependence of films with different central reflection wavelengths. For a locally planar cholesteric domain, combining $\lambda(\theta) = p\sqrt{\overline{n}^2 - n_{\mathrm{air}}^2 \sin^2\theta}$ with the normal-incidence relation $\lambda_0 = \overline{n}p$ gives the normalized angular response

$$\frac{\lambda(\theta)}{\lambda_0} = \sqrt{1 - \frac{n_{\mathrm{air}}^2}{\overline{n}^2} \sin^2\theta}. \quad (7)$$

For a locally planar cholesteric, normalization by $\lambda_0 = np$ removes the explicit dependence on pitch p, leaving an angular response determined by the refractive index n and the assumed helix-axis orientation. For the experimental comparison, the wavelength measured at the microscope-defined central angle, $\lambda(0^\circ)$, was used as the reference value.

The measured mean blue-shift magnitudes between $\theta = 0^\circ$ and $\pm 30^\circ$ are 33, 26, and 26 nm for the [8 J/mL, 0 mmol/kg], [8 J/mL, 150 mmol/kg], and FCD-rich [120 J/mL, 150 mmol/kg] films, respectively. The corresponding central reflection wavelengths, $\lambda(0^\circ)$, are 618, 563, and 774 nm. Normalization by these values gives fractional blue-shift magnitudes of 5.3, 4.7, and 3.3%, respectively. The FCD-rich film therefore exhibits the weakest normalized angular dispersion.

For the comparison shown in SI Figure S16a,c,e, the measured central wavelength $\lambda(0^\circ)$ was used as the reference value of λ_0. The calculated curves were generated using the refractive index of dry CNC films reported in the literature: $\overline{n} = 1.5$-1.6.[S4,S15] For each assumed value of $\overline{n}$, the pitch was set to $p = \lambda(0^\circ)/\overline{n}$. This procedure constrains the calculated response to the measured central wavelength and tests whether the subsequent angular dependence is consistent with the locally planar model.

For $n_{\text{air}} = 1$ and $\theta = 30^\circ$, Equation 7 predicts a fractional blue-shift magnitude of

$$|\delta_{\overline{n}=1.5}| = 1 - \sqrt{1 - \frac{\sin^2(30^\circ)}{1.5^2}} = 0.057, \tag{8}$$

or approximately 5.7%, for $\overline{n} = 1.5$. For $\overline{n} = 1.6$, the predicted value is

$$|\delta_{\overline{n}=1.6}| = 1 - \sqrt{1 - \frac{\sin^2(30^\circ)}{1.6^2}} = 0.050, \tag{9}$$

or approximately 5.0%. The locally planar model therefore predicts a fractional blue-shift magnitude of approximately 5.0-5.7% over this refractive-index range.

The measured mean fractional blue-shift magnitude of 5.3% for the [8 J/mL, 0 mmol/kg] film lies within this predicted range, consistent with the overlays in SI Figure S16a. The value of 4.7% measured for the [8 J/mL, 150 mmol/kg] film is slightly smaller than the predicted range, as also visible in SI Figure S16c. The value of 3.3% for the FCD-rich film is substantially smaller, and SI Figure S16e shows the largest deviation from the locally planar response.

Within the locally planar model, the effective value of $\overline{n}$ required to reproduce a measured normalized response at $\theta = 30^\circ$ can be estimated from

$$\overline{n}_{\text{eff}} = \frac{n_{\text{air}} \sin\theta}{\sqrt{1 - [\lambda(\theta)/\lambda_0]^2}}. \tag{10}$$

Using the measured mean fractional shifts at $\pm 30^\circ$ gives $\overline{n}_{\text{eff}} \approx 1.56$, 1.65, and 1.96 for the [8 J/mL, 0 mmol/kg], [8 J/mL, 150 mmol/kg], and FCD-rich films, respectively. These effective values indicate the value that a strictly planar model would require to reproduce each measured normalized response. The value for the [8 J/mL, 0 mmol/kg] film lies within the literature-constrained range, the value for the [8 J/mL, 150 mmol/kg] film is modestly higher, and the value required for the FCD-rich film is close to 2.

The average optical index was not measured independently and may vary among the films because of differences in CNC alignment, density, and local birefringence. However, an average optical index close to 2 lies well above the range reported for dry CNC films.[S4,S15] Variation in $\overline{n}$ alone is therefore unlikely to explain the weak normalized angular dependence of the FCD-rich film. The comparison does not establish that focal conic curvature is the only contribution to the measured response, but the deviation is consistent with averaging over a spatial distribution of local helix-axis orientations within the curved focal conic layers.

The SEM cross-sections support the morphological interpretation of the optical response but were not used to obtain independent pitch values. The apparent periodicity measured from a cross-section depends on the orientation of the fracture plane relative to the local helical axis. This geometric uncertainty is especially large for the strongly curved layers of the FCD-rich film. The SEM images are therefore used to qualitatively compare layer geometry across the films.

The FWHM values in SI Figure S16b,d,f show that the reduced angular dispersion of the FCD-rich film is not accompanied by the narrowest local spectral response. The FCD-rich film has a FWHM of 58 nm, comparable to the value of 57 nm measured for the [8 J/mL, 0 mmol/kg] film and larger than the value of 43 nm measured for the [8 J/mL, 150 mmol/kg] film. Its distinguishing optical characteristic is therefore the combination of a well-defined local selective-reflection peak with the weakest normalized angular dispersion among the three films analyzed.

The wavelength extent of the high-intensity region across the complete angle-resolved map provides information that is distinct from the FWHM measured at a single angle. This two-

dimensional spectral-angular footprint depends both on the local spectral width at each angle and on the displacement of the reflection band across the measured angular range. Consequently, a film can display a narrow spectrum at $\theta = 0^\circ$ while spanning a broader wavelength interval in the full map if its reflection maximum shifts strongly with angle. This behavior is observed for the [8 J/mL, 150 mmol/kg] film, which has the smallest fitted FWHM but retains a clear angular blue shift in SI Figure S16c,d.

In contrast, the high-intensity region of the FCD-rich film remains confined to a smaller wavelength range across the measured angles in SI Figure S16e, even though its spectrum at $\theta = 0^\circ$ is not the narrowest of the three films. This reduced spectral-angular footprint arises primarily from the weak displacement of the reflection maximum with angle and provides a direct visualization of its reduced iridescence.

The combination of a well-defined reflection band and a small spectral-angular footprint is consistent with the focal conic morphology. The curved layers provide a spatial distribution of local helix-axis orientations, so different portions of the structure can contribute to reflection as the external angle changes while remaining within a similar wavelength range. The optical response therefore suggests that the FCD-rich film combines locally retained cholesteric periodicity with an orientation distribution that reduces the angular displacement of the ensemble-averaged reflection band.

All angle-resolved spectroscopy maps

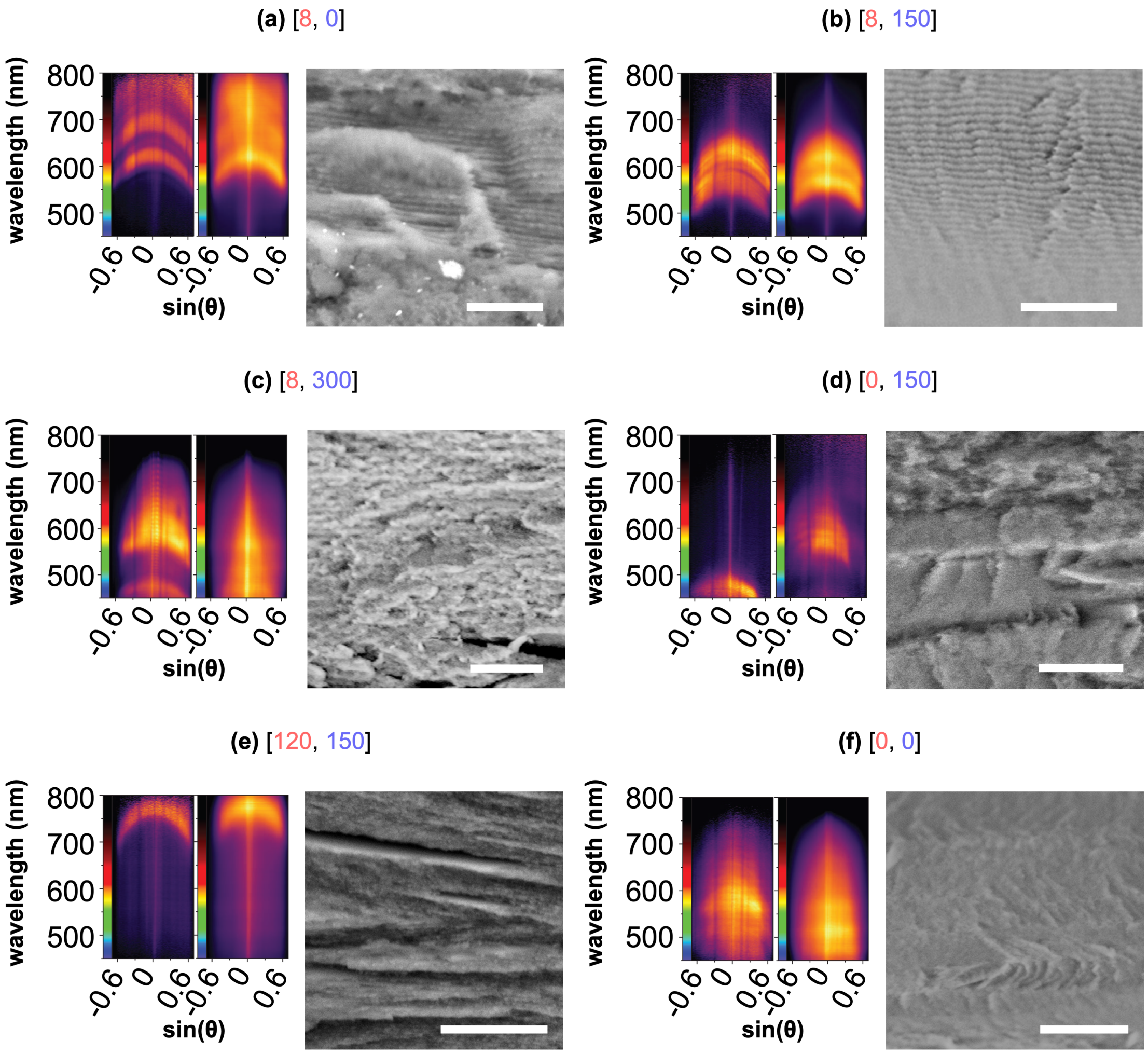

Figure S17: For each of six films (a-f), angle-resolved reflectance spectra were obtained for a small region (20 μm diameter, left) and a large region (200 μm diameter, right). The rightmost image for each film is its corresponding exposed cross-section taken with SEM. (a) [8 J/mL, 0 mmol/kg], (b) [8 J/mL, 150 mmol/kg], (c) [8 J/mL, 300 mmol/kg], (d) [0 J/mL, 150 mmol/kg], (e) [120 J/mL, 150 mmol/kg], (f) [0 J/mL, 0 mmol/kg]. SEM image scale bars: 10 μm.

Time-lapse videos

Time-lapse videos of different evaporating CNC suspensions were recorded with polarized optical microscopy. All of the videos provided are listed below.

- SI Video 1 (1m08s) - Evaporation time-lapse of sample [120 J/mL, 150 mmol/kg].
- SI Video 2 (1m39s) - Evaporation time-lapse of sample [120 J/mL, 150 mmol/kg] at a slowed evaporation rate. The video starts at the 188-hour mark (still in the isotropic regime).
- SI Video 3 (1m27s) - Evaporation time-lapse of sample [0 J/mL, 150 mmol/kg].
- SI Video 4 (1m07s) - Evaporation time-lapse of sample [8 J/mL, 150 mmol/kg].
- SI Video 5 (1m29s) - Evaporation time-lapse of sample [30 J/mL, 150 mmol/kg].
- SI Video 6 (0m56s) - Evaporation time-lapse of sample [0 J/mL, 0 mmol/kg].
- SI Video 7 (0m43s) - Evaporation time-lapse of sample [1440 J/mL, 0 mmol/kg].
- SI Video 8 (1m13s) - Evaporation time-lapse of sample [0 J/mL, 450 mmol/kg].

References

(S1) Girard, F.; Antoni, M.; Sefiane, K. On the Effect of Marangoni Flow on Evaporation Rates of Heated Water Drops. *Langmuir* **2008**, *24*, 9207–9210.

(S2) Gençer, A.; Schütz, C.; Thielemans, W. Influence of the Particle Concentration and Marangoni Flow on the Formation of Cellulose Nanocrystal Films. *Langmuir* **2017**, *33*, 228–234.

(S3) Shao, R.; Meng, X.; Shi, Z.; Zhong, J.; Cai, Z.; Hu, J.; Wang, X.; Chen, G.; Gao, S.; Song, Y.; Ye, C. Marangoni Flow Manipulated Concentric Assembly of Cellulose Nanocrystals. *Small Methods* **2021**, *5*, 2100690.

(S4) Dumanli, A. G.; van der Kooij, H. M.; Kamita, G.; Reisner, E.; Baumberg, J. J.; Steiner, U.; Vignolini, S. Digital Color in Cellulose Nanocrystal Films. *ACS Appl. Mater. Interfaces* **2014**, *6*, 12302–12306.

(S5) Hamad, W. Y.; Hu, T. Q. Structure–Process–Yield Interrelations in Nanocrystalline Cellulose Extraction. *Can. J. Chem. Eng.* **2010**, *88*, 392–402.

(S6) Beck, S.; Bouchard, J.; Berry, R. Controlling the Reflection Wavelength of Iridescent Solid Films of Nanocrystalline Cellulose. *Biomacromolecules* **2011**, *12*, 167–172.

(S7) Parton, T. G.; Parker, R. M.; van de Kerkhof, G. T.; Narkevicius, A.; Haataja, J. S.; Frka-Petesic, B.; Vignolini, S. Chiral Self-Assembly of Cellulose Nanocrystals Is Driven by Crystallite Bundles. *Nat. Commun.* **2022**, *13*, 2657.

(S8) Chen, Q.; Sriram, A.; Das, A.; Matic, K.; Hendija, M.; Tonry, K.; Ross, J. L.; Das, M.; McGorty, R. J.; Robertson-Anderson, R. M.; Valentine, M. T. BARCODE: High Throughput Screening and Analysis of Soft Active Materials. *Nat. Commun.* **2026**, *17*, 1192.

(S9) BARCODE: Biomaterial Activity Readouts to Categorize, Optimize, Design and Engineer. `https://github.com/BARCODE-HTP/barcode`, 2026; GitHub repository (accessed 2026-09-14).

(S10) Shafiei-Sabet, S.; Hamad, W. Y.; Hatzikiriakos, S. G. Rheology of Nanocrystalline Cellulose Aqueous Suspensions. *Langmuir* **2012**, *28*, 17124–17133.

(S11) Pal, R.; Pattath, K. Rheology of Suspensions Thickened by Cellulose Nanocrystals. *Nanomaterials* **2024**, *14*, 1122.

(S12) Wu, Q.; Li, X.; Li, Q.; Wang, S.; Luo, Y. Estimation of Aspect Ratio of Cellulose Nanocrystals by Viscosity Measurement: Influence of Aspect Ratio Distribution and Ionic Strength. *Polymers* **2019**, *11*, 781.

(S13) Beuguel, Q.; Tavares, J. R.; Carreau, P. J.; Heuzey, M.-C. Ultrasonication of Spray- and Freeze-Dried Cellulose Nanocrystals in Water. *J. Colloid Interface Sci.* **2018**, *516*, 23–33.

(S14) Bird, R. B.; Armstrong, R. C.; Hassager, O. *Dynamics of Polymeric Liquids, Volume 1: Fluid Mechanics*, 2nd ed.; Wiley: New York, 1987.

(S15) Frka-Petesic, B.; Kamita, G.; Guidetti, G.; Vignolini, S. Angular Optical Response of Cellulose Nanocrystal Films Explained by the Distortion of the Arrested Suspension upon Drying. *Phys. Rev. Mater.* **2019**, *3*, 045601.
